# Assessment of Coronary Inflammatory Risk From Noncontrast Cardiac Computed Tomography

**DOI:** 10.1001/jamacardio.2026.5006

**Published:** 2026-11-11

**Authors:** Kenneth Chan, Mark Colin Gissler, Ikboljon Sobirov, Elizabeth Wahome, Pete Tomlins, Laura V. Klüner, Evangelos K. Oikonomou, Edward Nicol, Jonathan Weir-McCall, Ronak Rajani, David Adlam, Jonathan Rodrigues, Nicholas Screaton, Attila Kardos, John P. Greenwood, Nikant Sabharwal, Andrew Kelion, Francesca Pugliese, Steffen E. Petersen, Vinoda Sharma, Vijay Kunadian, Timothy Fairbairn, Leanne Eveson, Michael Petrus, Stavros Spyropoulos, Christos P. Kotanidis, Alexios S. Antonopoulos, Chris Mathers, Muhammad Siddique, Sam Fry, Parijat Patel, Henry West, Ahmad Abdullrahman, Damaris Darby, Yogesh Sohan, Cheerag Shirodaria, Brittany Weber, Daniel Huck, Ron Blankstein, Nehal N. Mehta, Milind Desai, Bernard J. Gersh, Stephan Achenbach, Peter Libby, Stefan Neubauer, Keith M. Channon, John Deanfield, Charalambos Antoniades

**Affiliations:** 1 Acute Multidisciplinary Imaging and Interventional Centre, British Heart Foundation Centre of Research Excellence, Division of Cardiovascular Medicine, Radcliffe Department of Medicine, University of Oxford, Oxford, United Kingdom; 2 National Institute for Health and Care Research (NIHR) Oxford Biomedical Research Centre, Oxford University Hospitals NHS Foundation Trust, John Radcliffe Hospital, Oxford, United Kingdom; 3 Cardiology and Angiology, Medical Center–University of Freiburg, Faculty of Medicine, University of Freiburg, Freiburg, Germany; 4 Caristo Diagnostics Ltd, Oxford, United Kingdom; 5 Section of Cardiovascular Medicine, Department of Internal Medicine, Yale School of Medicine, New Haven, Connecticut; 6 Royal Brompton and Harefield Hospitals, London, United Kingdom; 7 School of Biomedical Engineering and Imaging Sciences, King’s College, London, United Kingdom; 8 Department of Cardiology, Guys and St Thomas’ Hospital, London, United Kingdom; 9 Department of Cardiovascular Sciences, University of Leicester and NIHR Leicester, Leicester, United Kingdom; 10 Leicester British Heart Foundation Centre of Research Excellence, Leicester, United Kingdom; 11 Leicester NIHR Biomedical Research Centre, Leicester, United Kingdom; 12 Royal United Hospitals Bath NHS Foundation Trust, Bath, United Kingdom; 13 Royal Papworth Hospital, Cambridge, United Kingdom; 14 Department of Cardiology, Translational Cardiovascular Research Group, Milton Keynes University Hospital NHS Foundation Trust, Milton Keynes, United Kingdom; 15 Baker Heart and Diabetes Institute, Melbourne, Victoria, Australia; 16 Barts Heart Centre, St Bartholomew’s Hospital, Barts Health NHS Trust, London, United Kingdom; 17 William Harvey Research Institute, Barts and The London School of Medicine and Dentistry, Queen Mary University of London, London, United Kingdom; 18 Department of Cardiology, Sandwell and West Birmingham NHS Trust, Birmingham, United Kingdom; 19 Cardiovascular Science, University of Birmingham, Birmingham, United Kingdom; 20 Translational and Clinical Research Institute, Faculty of Medical Sciences, Newcastle University, Newcastle upon Tyne, United Kingdom; 21 Cardiothoracic Centre, Freeman Hospital, Newcastle upon Tyne Hospitals NHS Foundations Trust, Newcastle upon Tyne, United Kingdom; 22 Liverpool Heart and Chest Hospital NHS Foundation Trust, Liverpool, United Kingdom; 23 Heart and Vascular Institute and Center for Inflammation Imaging, Mass General Brigham and Harvard Medical School, Boston, Massachusetts; 24 Division of Cardiology, Department of Medicine University of Texas Southwestern, Dallas; 25 Department of Dermatology, UT Southwestern Medical Center, Dallas, Texas; 26 The George Washington University School of Medicine and Health Sciences, Washington, DC; 27 Cleveland Clinic Heart and Vascular Institute, Cleveland, Ohio; 28 Department of Cardiovascular Medicine, Mayo Clinic College of Medicine and Science, Rochester, Minnesota; 29 Department of Cardiology, Friedrich-Alexander-University Erlangen-Nürnberg, Erlangen, Germany; 30 Institute of Cardiovascular Science, University College London, London, United Kingdom

## Abstract

**Importance:**

Individuals with low or minimal coronary artery calcification (CAC) are considered at low cardiovascular risk, but a substantial absolute number of cardiovascular events occur in this population; assessment of coronary inflammation using the fat attenuation index (FAI) score may identify residual inflammatory risk beyond CAC score.

**Objective:**

To assess whether coronary inflammation measured on coronary computed tomography angiography (CCTA) or noncontrast cardiac CT (NCCT) identifies individuals at high cardiovascular risk independent of CAC burden.

**Design, Setting, and Participants:**

In this cohort study, a novel artificial intelligence algorithm for measuring FAI score on NCCT was trained and tested in an independent, multicenter, longitudinal cohort of consecutive individuals undergoing clinically indicated CCTA and NCCT for CAC score between 2010 and 2021, with median (IQR) follow-up of 6.7 (4.6-10.0) years. Data were analyzed from January 2010 to March 2024.

**Exposure:**

Agatston CAC score and coronary inflammation from both CCTA (CCTA FAI score, established biomarker) and NCCT (NC FAI score, novel biomarker).

**Main Outcomes and Measures:**

The primary outcome was cardiovascular (CV) mortality; the secondary outcome was major adverse cardiovascular events (MACE), comprising myocardial infarction, new-onset heart failure, or CV death.

**Results:**

The derivation cohort included 6818 individuals (median [IQR] age, 61 [51-71] years; 3543 female participants [52.0%]), and the external validation clinical outcomes cohort included 19 110 individuals (median [IQR] age, 56.0 [48.0-64.0] years; 9289 female participants [48.6%]). During follow-up, 536 CV deaths and 1727 MACE occurred. FAI scores independently stratified CV mortality risk across CAC score categories, with the strongest associations among individuals with CAC score of 0. Adding CCTA FAI score or NC FAI score to CAC score significantly improved risk discrimination (area under the curve increased from 0.76 to 0.81 and 0.80, respectively; *P* < .001), reclassification (net reclassification index), and net benefit across all risk thresholds. Among 89 CV deaths in individuals with CAC score of 0 and 138 CV deaths with CAC score of 1 through 99, 44.9% and 44.2% had NC FAI score above the 75th percentile, respectively. Among individuals with CAC score of 0, CV mortality rates increased from 1.4 to 4.0 per 1000 person-years for those with NC FAI score above the 75th percentile, and MACE rates increased from 5.9 to 13.3 per 1000 person-years.

**Conclusions and Relevance:**

Per the results of this cohort study, NC FAI score provides prognostic performance comparable to CCTA FAI score and improves cardiovascular risk prediction and reclassification beyond CAC score. Elevated FAI score, derived from either CCTA or NCCT, should be interpreted as a risk-enhancing factor that may prompt intensification of established, evidence-based preventive measures and may serve as a potential future gatekeeper for the use of targeted anti-inflammatory treatments.

## Key Points

**Question:**

Can coronary inflammation be quantified on noncontrast computed tomography (CT) performed for coronary artery calcium (CAC) scoring and identify high-risk individuals with zero or minimal CAC who are otherwise considered at low risk?

**Findings:**

A substantial proportion of all cardiovascular events occur among individuals with CAC score less than 100 Agatston units (42% for cardiovascular mortality and 51% for major adverse cardiovascular events). Coronary inflammation quantified by fat attenuation index (FAI) score on contrast coronary CT angiography or noncontrast CT improves cardiovascular risk prediction and reclassification beyond CAC score even among individuals with CAC score of zero.

**Meaning:**

Assessment of coronary inflammation from routine noncontrast CAC scans enables scalable risk stratification beyond CAC score alone, identifying residual inflammatory risk among individuals with zero or minimal CAC and enabling more personalized allocation of inflammation-targeted preventive therapies.

## Teaser

This cohort study assesses whether coronary inflammation measured on coronary computed tomography (CT) angiography or noncontrast cardiac CT identifies individuals at high cardiovascular risk independent of coronary artery calcification burden.

## Introduction

Coronary artery calcification (CAC) is routinely quantified on noncontrast computed tomography (CT) scans as a surrogate marker of atherosclerotic burden.^1^ The Agatston CAC score has been extensively validated as an independent predictor of cardiovascular risk^2^ and is recommended by clinical guidelines for cardiovascular prevention.^5^ More than half of individuals with suspected coronary artery disease (CAD) undergoing coronary CT angiography (CCTA) have no detectable CAC,^8^ contributing to the high negative predictive value of CCTA for future major adverse cardiovascular events (MACE).^9^ Thus, zero CAC score is often deemed reassuring and leads to deferral of statin therapy in those at low or borderline risk.^1^ However, cardiovascular events frequently arise from the rupture of inflamed, noncalcified plaques, which are not visualized on noncontrast cardiac CT,^12^ whereas calcified plaques are generally considered to be stable.^14^ Consequently, absent or low CAC does not preclude clinically important residual cardiovascular risk. In a large cohort of asymptomatic individuals followed up for over a decade, 42.8% of all cardiovascular deaths occurred in those with a CAC of zero at the time of the index scan and 33.3% in those with CAC of 1 through 100.^16^ Thus, despite widespread use of CAC score in risk reclassification and its incorporation into the 2026 dyslipidemia guideline to inform preventive treatment decisions,^11^ substantial unmet needs remain to identify higher-risk individuals within the large population with absent or minimal CAC who would otherwise be classified as low risk.

Coronary inflammation drives both atherogenesis and atherosclerotic plaque rupture,^17^ where identifying individuals with high coronary inflammation can add significant value to risk stratification.^18^ We previously demonstrated that coronary inflammation drives changes in the perivascular adipose tissue (PVAT) that can be quantified on routine CCTA by measuring the perivascular fat attenuation index (FAI) score,^19^ a standardized, generalizable, and US Food and Drug Administration (FDA)–approved surrogate measure of coronary inflammation with significant prognostic value.^12^ The use of the software-as-a-service (SAAS) model enables easy access to this tool as cloud-based technology in the clinic, allowing its broader use as a standardized medical device. However, the role of FAI score in individuals with zero or low CAC remains unclear. Its application in primary prevention is also constrained by the need for contrast-enhanced CCTA to delineate the coronary arterial wall, whereas CAC assessment is typically performed using low-dose, noncontrast cardiac CT. With the advent of novel anti-inflammatory treatments, detecting residual inflammatory risk from the same scans used for CAC scoring could transform cardiovascular prevention.

In this cohort study, we tested whether FAI score derived from CCTA (CCTA FAI score) adds prognostic value beyond CAC scoring, particularly among those individuals with low or zero CAC score. We then present a novel algorithm for measuring FAI score from noncontrast CT (NC FAI score) and evaluate its prognostic value beyond CAC score measured on the same scans.

## Methods

### Study Participants

The Oxford Risk Factor and Non-Invasive Imaging (ORFAN) study is a multicenter longitudinal cohort study of patients with a clinical indication for CCTA (ClinicalTrials.gov identifier: NCT05169333). Its design, patient selection, and data linkage have been described previously and are also presented in the eAppendix in Supplement 1.^12^ This study included consecutive individuals who underwent paired noncontrast CT for CAC score and CCTA as part of routine clinical care.

The derivation cohort for developing FAI score algorithm from noncontrast cardiac CT included 6818 participants from 3 hospitals in the Midlands or Northern England (Liverpool Heart and Chest Hospital, Milton Keynes Hospital, and Newcastle Hospitals). The external validation clinical outcomes cohort included individuals from 10 geographically separate hospitals across the UK who underwent clinically indicated CAC scoring and CCTA for suspected CAD between 2010 and 2021. Patients undergoing CT imaging for the evaluation of congenital heart disease or heart transplant were excluded. These individuals were followed up prospectively for a median (IQR) of 6.7 (4.6-10.0) years (eFigure 1 in Supplement 1). Demographic data were obtained from local electronic health record databases and National Health Service (NHS) England records. Imaging data were transferred to the ORFAN core laboratory at the University of Oxford via a Health Insurance Portability and Accountability Act (HIPAA)– and General Data Protection Regulation (GDPR)–compliant gateway. The program is sponsored by the University of Oxford and is governed by a Publications Committee that oversees publications and data sharing. The imaging core laboratory operates under a university-regulated quality management system, and statistical analyses were conducted within a secure data environment by the ORFAN statistical team, blinded to image analysis. Patient data were collected under Section 251 of the NHS Act 2006 with a waiver of informed consent with Confidentiality Advisory Group approval (20/CAG/0157) and approved by the Oxfordshire Research Ethics Committee (REC 15/SC/0545). Data reporting conforms to the Strengthening the Reporting of Observational Studies in Epidemiology (STROBE) reporting guideline.

### Clinical Events

Participants were followed up from the time of the CT scan until an event or until the end of the follow-up period on March 31, 2024, whichever occurred first. Clinical outcomes data were obtained from nationwide databases (cross-referenced between NHS Digital, the National Institute of Cardiovascular Outcomes Research, the Office for National Statistics, and the local electronic patient records, as described in the eAppendix in Supplement 1) and were reviewed by the ORFAN Events Adjudication team according to the study protocol. The primary end point was cardiovascular mortality, and the secondary was the composite end point of MACE, including myocardial infarction (MI), new-onset heart failure (HF), and cardiovascular mortality. Definitions of clinical end points are detailed in eTable 1 in Supplement 1.

### Quantification of CAC and Coronary Inflammation

Cardiac imaging and image analysis pipelines have been presented previously.^12^ Contrast-enhanced CCTAs were performed as per clinical protocol, optimized with the administration of β-blockers and sublingual glyceryl trinitrate (eAppendix in Supplement 1). After transfer to the ORFAN core laboratory, CCTA scans were analyzed using an unsupervised deep learning model within the CaRi-Heart toolkit (Caristo Diagnostics) to derive the FAI score for each coronary artery.^21^ The mean FAI score was calculated as the average of the 3 coronary artery scores. CAC scores were derived from noncontrast electrocardiogram-gated cardiac CT scans,^3^ and the Agatston CAC score of the whole coronary tree was extracted from clinical reports. CAC scores were categorized into the following risk groups: 0 (absent), 1 to 99 (mild), 100 to 299 (moderate), and more than 300 (severe) Agatston units.

Development of FAI score from noncontrast cardiac images (NC FAI score) is detailed in the eAppendix in Supplement 1. Briefly, dedicated deep learning–enabled segmentation and analysis were developed for NCCT in the derivation cohort, guided by artificial generative contrast on the noncontrast CT phase to help delineate lumen and PVAT interface. The FAI score values from the NCCT were then calculated as previously described using a research version of the CaRi-Heart medical device, developed for this purpose.

### Statistical Analysis

Patient characteristics were compared using Pearson χ^2^ for categorical variables and *t* tests and analysis of variance (ANOVA, 3 groups) for continuous variables, as appropriate. For survival analysis, individual follow-up time was calculated from the CCTA date until the first MACE occurrence or the last date of data extraction (March 31, 2024), whichever occurred first. Failure probabilities since CT scan were estimated and plotted using Kaplan-Meier curves. A Cox regression model was fitted to estimate the hazard ratios (HRs) and the 95% confidence intervals. Using time-dependent receiver operating characteristic (ROC) curve analysis, we calculated the area under the curve (AUC) to assess model discrimination in predicting cardiovascular events. Decision curve analysis was performed to evaluate the net benefit of integrating FAI score to CAC score, and we compared these with alternative strategies (eg, assuming that all patients were treated or nobody was treated). Reclassification in risk prediction was evaluated using continuous net reclassification improvement, with 95% confidence intervals derived from 200 bootstrap replications over a 5-year horizon. Multivariable analysis of the FAI score was performed using Cox regression models adjusted for cardiovascular risk factors. Alternative survival models using the Fine-Gray method were constructed to account for competing risk from noncardiovascular mortality. Schoenfeld residuals plots visually assessed the proportional hazards assumptions. There were minimal to no violations of the assumptions for all the events. Statistical tests were performed using Stata version 18.0 (StataCorp) and the R environment (R version 4.5.2 [The R Foundation for Statistical Computing]) using R Studio version 2026.01.1 with the following packages: rms, survival, timeROC, and survivalROC. All tests were 2-sided, and values of *P* < .05 were considered statistically significant.

## Results

### Study Cohorts and Clinical Outcomes

Baseline characteristics for the derivation cohort are presented in eTable 2 in Supplement 1, and for the external validation longitudinal cohort, in the Table. Briefly, the derivation cohort included 6818 individuals (median [IQR] age, 61 [51-71] years; 3543 female participants [52.0%]), and the external validation clinical outcomes cohort included 19 110 individuals (median [IQR] age, 56.0 [48.0-64.0] years; 9289 female participants [48.6%]). Approximately half of the population in the external validation cohort had a CAC score of zero (8651 patients [45.2%]), and 5963 (31.2%) had a CAC score of 1 through 99. A total of 536 cardiovascular deaths and 1727 MACE occurred during follow-up. Of these, 227 cardiovascular deaths and 873 MACE occurred among individuals with a CAC score less than 100, accounting for 42.3% and 50.8% of all cardiovascular mortality and MACE events, respectively (Table). Importantly, 89 cardiovascular deaths and 380 MACE occurred among individuals with a CAC score of zero, accounting for 16.6% and 22.0% of all cardiovascular mortality and MACE events, respectively.

**Table.** Baseline Characteristics and Major Adverse Cardiac Events of the External Validation Cohort

Characteristic or outcome	No. (%)					*P* value
	All participants (n = 19 110)	CAC 0 (n = 8651)	CAC 1-99 (n = 5963)	CAC 100-299 (n = 2080)	CAC ≥300 (n = 2416)	
Demographics						
Age at scan, median (IQR), y	56.0 (48.0-64.0)	51.0 (44.0-58.0)	58.0 (51.0-65.0)	62.0 (56.0-69.0)	67.0 (59.0-74.0)	<.001
Sex						
Female	9289 (48.6)	4925 (56.9)	2709 (45.4)	866 (41.6)	789 (32.7)	<.001
Male	9821 (51.4)	3726 (43.1)	3254 (54.6)	1214 (58.4)	1627 (67.3)	
Ethnicity^a^						
Asian	2827 (14.8)	1410 (16.3)	874 (14.7)	276 (13.3)	267 (11.1)	<.001
Black	1193 (6.2)	750 (8.7)	306 (5.1)	58 (2.8)	79 (3.3)	
White	7720 (40.4)	2868 (33.2)	2532 (42.5)	1044 (50.2)	1276 (52.8)	
Multiracial	252 (1.3)	121 (1.4)	68 (1.1)	30 (1.4)	33 (1.4)	
Other groups^b^	6641 (34.8)	3254 (37.6)	2029 (34.0)	634 (30.5)	724 (30.0)	
Unknown	477 (2.5)	248 (2.9)	154 (2.6)	38 (1.8)	37 (1.5)	
Risk factors						
Hypertension	7797 (40.8)	2616 (30.2)	2601 (43.6)	1078 (51.8)	1502 (62.2)	<.001
Hyperlipidemia	6770 (35.4)	2525 (29.2)	2285 (38.3)	855 (41.1)	1105 (45.7)	<.001
Type 2 diabetes	2336 (12.2)	690 (8.0)	743 (12.5)	358 (17.2)	545 (22.6)	<.001
Smoking status						
Nonsmoker	14 407 (75.4)	6757 (78.1)	4511 (75.6)	1513 (72.7)	1626 (67.3)	<.001
Former smoker	2455 (12.8)	837 (9.7)	750 (12.6)	325 (15.6)	543 (22.5)	
Current smoker	2248 (11.8)	1057 (12.2)	702 (11.8)	242 (11.6)	247 (10.2)	
Medications						
Antiplatelets	7323 (38.3)	1766 (20.4)	2755 (46.2)	1229 (59.1)	1573 (65.1)	<.001
β-Blockers	8209 (43.0)	2796 (32.3)	2677 (44.9)	1207 (58.0)	1529 (63.3)	<.001
Calcium channel blockers	7111 (37.2)	2746 (31.7)	2431 (40.8)	877 (42.2)	1057 (43.8)	<.001
Statins	4179 (21.9)	1384 (16.0)	1493 (25.0)	603 (29.0)	699 (28.9)	<.001
ACE inhibitors	6257 (32.7)	2230 (25.8)	2113 (35.4)	879 (42.3)	1035 (42.8)	<.001
Angiotensin receptor blockers	186 (1.0)	68 (0.8)	53 (0.9)	33 (1.6)	32 (1.3)	<.001
Diuretics	3882 (20.3)	1180 (13.6)	1260 (21.1)	587 (28.2)	855 (35.4)	<.001
Scanner vendors						
GE Medical Systems	993 (5.2)	363 (4.2)	320 (5.4)	133 (6.4)	177 (7.3)	<.001
Siemens	16 969 (88.8)	7960 (92.0)	5246 (88.0)	1835 (88.2)	1988 (82.3)	
Philips	73 (0.4)	45 (0.5)	15 (0.3)	9 (0.4)	4 (0.2)	
Canon	1075 (5.6)	283 (3.3)	382 (6.4)	163 (7.8)	247 (10.2)	
Outcomes						
Cardiovascular death	536 (2.8)	89 (1.0)	138 (2.3)	83 (4.0)	226 (9.4)	<.001
Fatal or nonfatal myocardial infarction	877 (4.6)	186 (2.2)	260 (4.4)	159 (7.6)	272 (11.3)	<.001
New-onset heart failure	704 (3.7)	160 (1.9)	195 (3.3)	131 (6.3)	218 (9.0)	<.001
Major adverse cardiovascular events	1727 (9.0)	380 (4.4)	493 (8.3)	298 (14.3)	556 (23.0)	<.001

Abbreviations: ACE, angiotensin-converting enzyme; CAC, coronary artery calcium.

^a^ Self-reported.

^b^ Self-reported "any other ethnicity" in the national registry.

### Interplay of Coronary Calcification and Inflammation for Prediction of Cardiovascular Outcomes

In the external validation cohort, FAI score measured from contrast (CCTA FAI score) or noncontrast (NC FAI score) showed good correlation and agreement across all 3 coronary arteries (eFigure 2 in Supplement 1). CAC score showed a modest but statistically significant correlation with mean CCTA FAI score (Spearman ρ, 0.25; *P* < .001), as well as mean NC FAI score (Spearman ρ, 0.21; *P* < .001). FAI score was independently predictive of cardiovascular mortality after adjusting for CAC score, regardless of whether it was measured on CCTA or NCCT (Figure 1A and B) (CCTA FAI score: HR, 8.01; 95% CI, 6.01-10.7; *P* < .001; NC FAI score: HR, 6.53; 95% CI, 4.94-8.64; *P* < .001 comparing those with ≥90th percentile to <25th percentile). The findings were consistent for FAI scores measured in each individual coronary artery (eTable 3 in Supplement 1). After further adjusting for clinical cardiovascular risk factors, CCTA FAI score and NC FAI score remained independently prognostic of individual secondary clinical end points (MI, new-onset HF) and composite MACE outcomes (eTables 3 and 4 in Supplement 1). Sensitivity analyses accounting for competing risk of noncardiovascular deaths using Fine-Gray subdistribution hazard models showed minimal impact on the results (eTable 5 in Supplement 1). Similar findings were observed in the subgroup of individuals with no known atherosclerotic cardiovascular disease prior to CT imaging (eFigure 3 in Supplement 1). In the ROC analyses, CAC score showed good performance in discriminating cardiovascular death, with an AUC of 0.76 for cardiovascular mortality (Figure 1D). The AUC NC FAI score or CCTA FAI score alone for discriminating cardiovascular mortality was 0.75 and 0.77, respectively. Adding NC FAI score or CCTA FAI score to CAC score significantly improved the risk discrimination to 0.80 (Δ AUC, 0.04; *P* < .001) and 0.81 (Δ AUC, 0.05; *P* < .001), respectively (Figure 1D). There was no statistically significant difference in the AUC between the model using NC FAI score vs CCTA FAI score (Figure 1C and D). The addition of NC FAI score or CCTA FAI score to quantitative plaque volumes (calcified and noncalcified plaques) significantly improved the AUC across the 10-year horizon (eFigure 4 in Supplement 1). Furthermore, decision curve analysis showed a significant net benefit of adding NC FAI score or CCTA FAI score to CAC score across the clinically relevant risk thresholds (eFigure 5 in Supplement 1). These results demonstrate the value of assessing coronary inflammation beyond coronary calcification alone.

**Figure 1.**
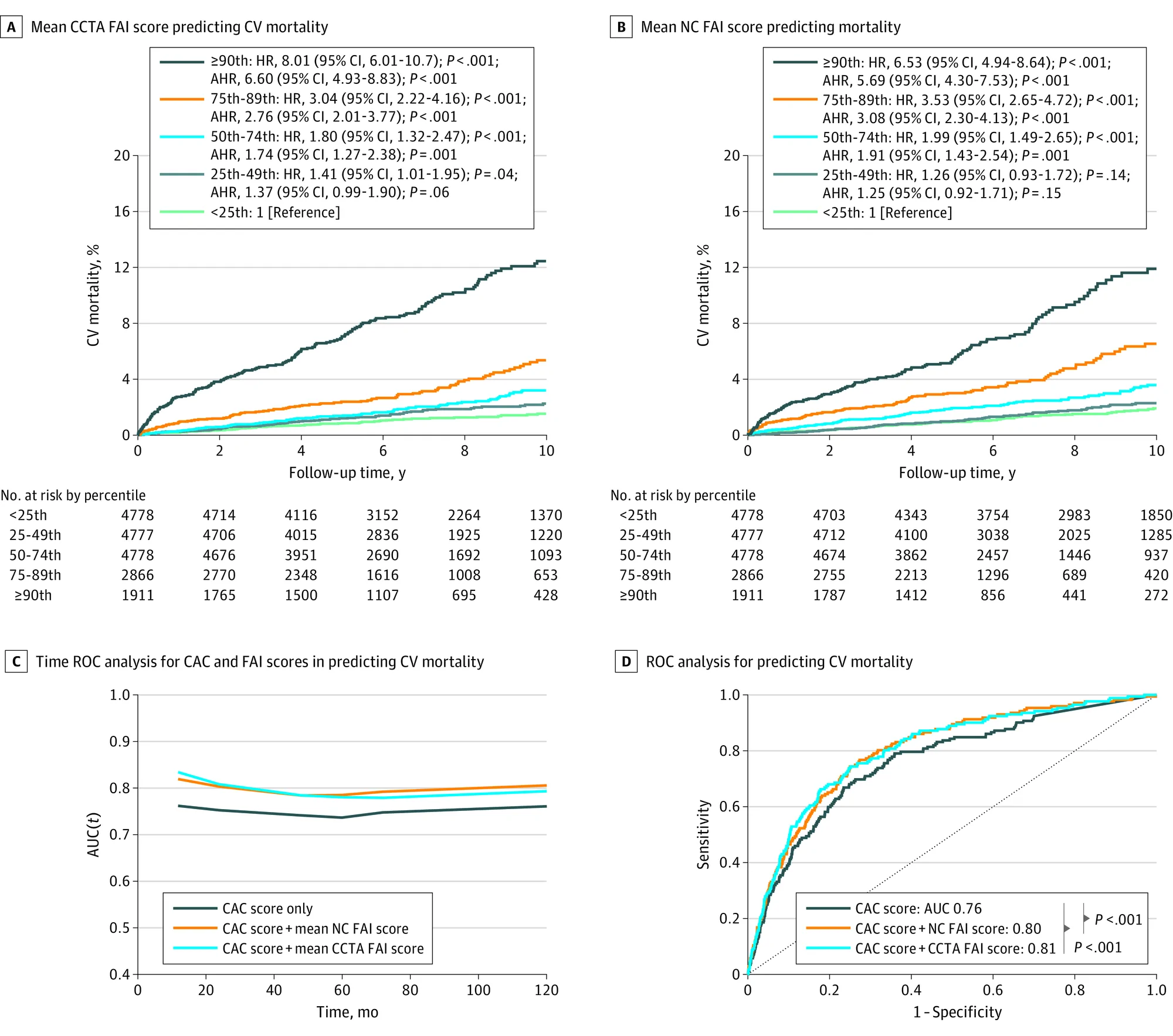
Kaplan-Meier Plots Showing Independent Prognostic Value of Coronary Inflammation Beyond Coronary Calcification for Cardiovascular Risk Stratification A and B, Kaplan-Meier curves for the prognostic value of mean fat attenuation index score derived from coronary computed tomography angiography (CCTA FAI score) and FAI score derived from noncontrast CT (NC FAI score) for cardiovascular (CV) mortality in the whole study population. C and D, Time-dependent receiver operating characteristic (ROC) analysis, ROC curves, and area under the curve (AUC) values for prediction of CV mortality by coronary artery calcification (CAC) score (dark blue), CAC score + mean CCTA FAI score (light blue), or CAC score + mean NC FAI score (orange). AHR indicates hazard ratios after adjustment for CAC score.

### Risk Stratification and Reclassification Among Individuals With No or Minimal Coronary Calcification

NC FAI score and CCTA FAI score both significantly stratified the risk for cardiovascular mortality across all CAC score and coronary stenosis categories, independently from age, sex, and traditional cardiovascular risk factors (Figure 2). Notably, the strongest prognostic value of both CCTA FAI score and NC FAI score for cardiovascular mortality was observed among individuals with CAC score of 0 (HR per SD, 1.54; 95% CI, 1.40-1.70; *P* < .001; and HR per SD, 1.59; 95% CI, 1.40-1.81, respectively). Similar findings were observed for predicting MACE (eFigure 6 in Supplement 1). Among individuals with CAC score of 0, CV mortality rates increased from 1.4 to 4.0 per 1000 person-years for those with NC FAI score above the 75th percentile, and MACE rates increased from 5.9 to 13.3 per 1000 person-years. Furthermore, both CCTA FAI score and NC FAI score significantly stratified the cardiovascular risk among individuals with no plaques (ie, absence of calcified and noncalcified plaques) (eFigure 7 in Supplement 1). Here, the net reclassification index significantly improved for CCTA FAI score (0.29; 95% CI, 0.22-0.34) and NC FAI score (0.29; 95% CI, 0.23-0.35) (eFigure 8 in Supplement 1). Among individuals with a CAC score of 0, FAI score correctly reclassified 46.1% (CCTA FAI score) and 44.9% (NC FAI score) of cardiovascular mortality events into the high- or very high–risk category (Figure 3; eTable 6 in Supplement 1). Among individuals with CAC scores of 1 through 99, 45.7% and 44.2% cardiovascular mortality events were reclassified as high or very high risk by CCTA FAI score and NC FAI score, respectively (Figure 3; eTable 6 in Supplement 1). Subgroup analysis demonstrated that both CCTA FAI score and NC FAI score significantly stratified the risk of cardiovascular mortality across age, sex, and presence or absence of traditional cardiovascular risk factors (Figure 4).

**Figure 2.**
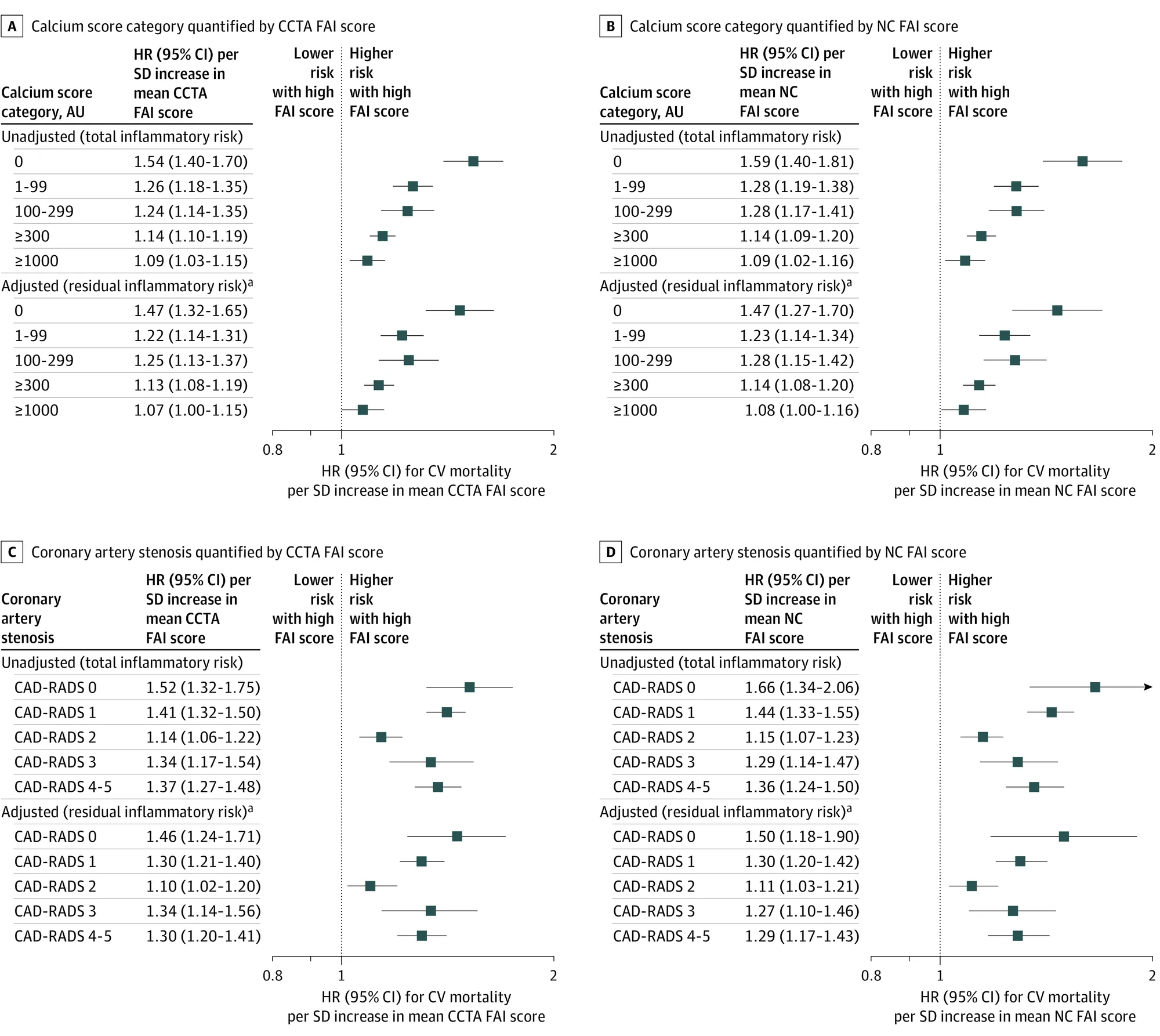
Forest Plots Showing Predictive Value of Coronary Inflammation at Different Stages of Coronary Calcification and Stenosis Coronary inflammation quantified by mean fat attenuation index (FAI) score from coronary computed tomography angiography (CCTA) (A, C) and mean FAI score from noncontrast CT (B, D) as a predictor of cardiovascular (CV) mortality in patients with different levels of coronary artery calcification (CAC) defined by CAC score categories and at different levels of coronary artery stenosis defined by Coronary Artery Disease–Reporting and Data System 2.0 (CAD-RADS 2.0) categories. AU indicates Agatston units; HR, hazard ratio. ^a^Adjusted for age, sex, hypertension, type 2 diabetes, hyperlipidemia, and smoking status.

**Figure 3.**
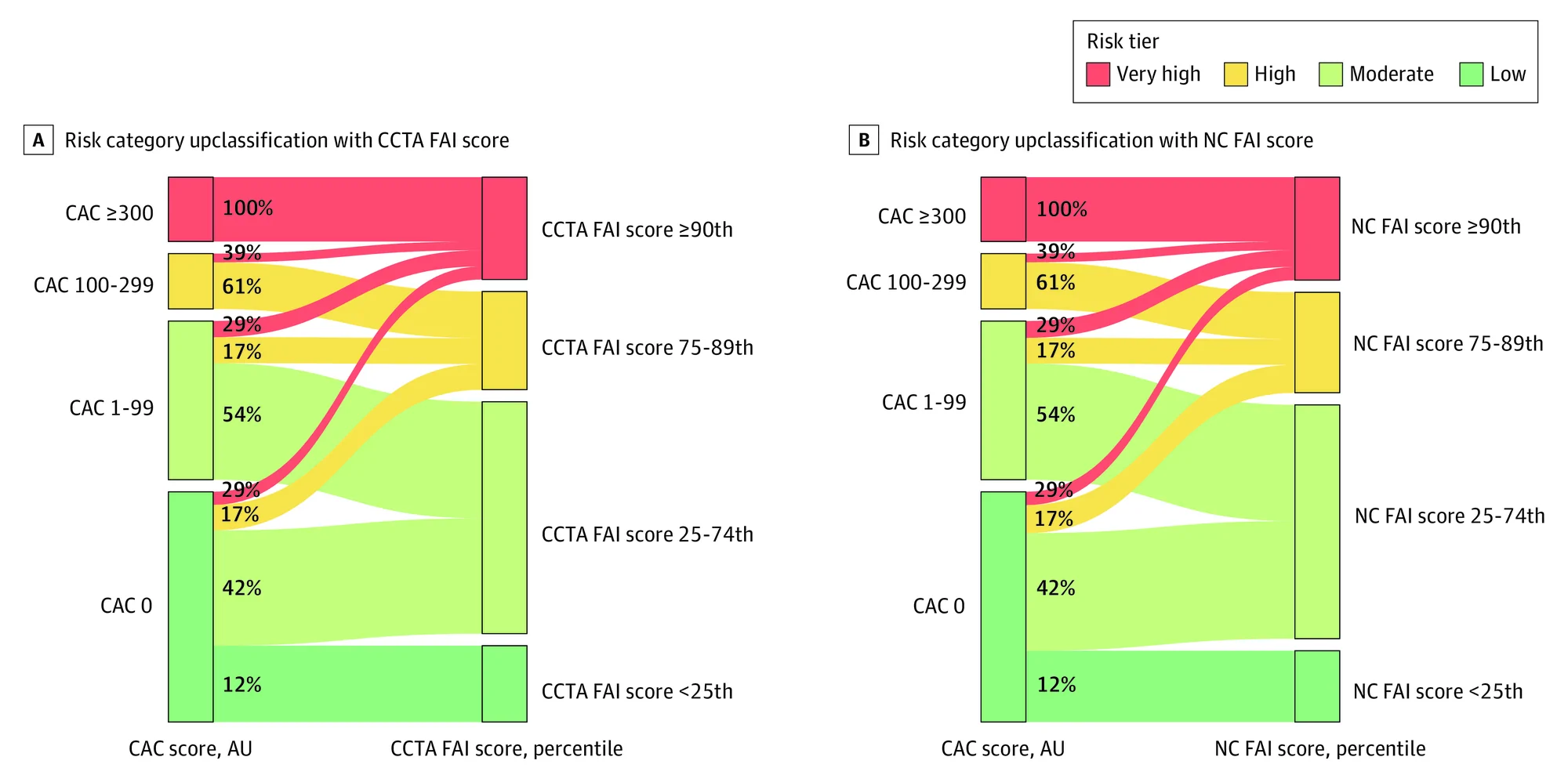
Sankey Diagram Showing Risk Reclassification Analysis for Probability of Cardiovascular Mortality Sankey diagram displaying upward reclassification of coronary artery calcification (CAC) risk categories by addition of fat attenuation index score measured from coronary computed tomography angiography (CCTA FAI score, A) or FAI score measured from noncontrast CT (NC FAI score, B). Percentages indicate CV mortality events in each CAC risk category. AU indicates Agatston units.

**Figure 4.**
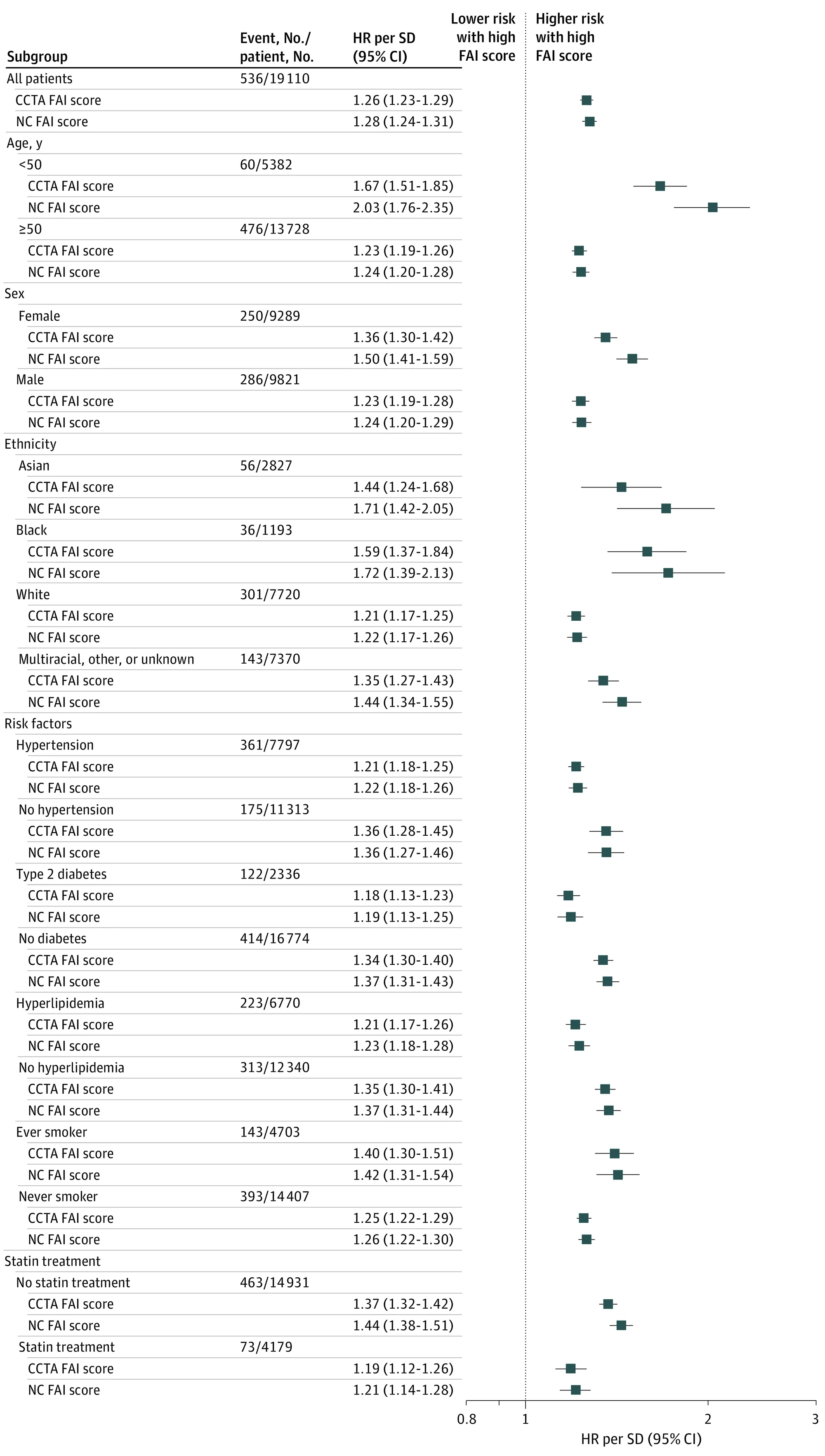
Forest Plot Showing Subgroup Analysis of the Predictive Value of Fat Attenuation Index Score Measured From Coronary Computed Tomography Angiography (CCTA FAI Score) and FAI Score Measured From Noncontrast CT (NC FAI Score) for Cardiovascular Mortality Forest plot showing hazard ratios (HRs) per SD increase in mean NC FAI score for cardiovascular mortality over a 10-year follow-up period in individual subgroups. Ethnicity was self-reported, and the “other” category includes those who self-reported "any other ethnicity" in the national registry.

## Discussion

This cohort study demonstrates that the NC FAI score derived from noncontrast CAC scans provides prognostic value comparable to CCTA FAI score, with both metrics improving cardiovascular risk prediction beyond CAC. The prognostic value of FAI score was greatest in individuals with absent or minimal calcification and was independent of age, sex, and clinical risk factors. Among individuals with zero CAC, elevated FAI score identified a subgroup with cardiovascular mortality exceeding contemporary guideline thresholds for preventive pharmacotherapy. These findings suggest the existence of a zero calcium but high coronary inflammation high-risk group, where assessment of coronary inflammation can uncover residual risk not captured by the CAC score alone and could help refine preventive treatment decisions.

CAC scoring has transformed cardiovascular risk assessment by providing a reproducible measure of established coronary atherosclerotic burden^22^ and is widely endorsed by clinical guidelines to guide preventive therapies.^11^ Higher CAC scores reflect greater calcified plaque burden and provide prognostic information beyond traditional risk factors.^4^ However, the power of zero CAC does not imply the absence of risk. Given the large population with CAC zero, a substantial absolute number of cardiovascular events occur in this group, with a recent report suggesting that approximately 43% of fatal cardiovascular events occur among individuals with zero CAC.^25^ In our study, NC FAI score above the 75th percentile identified nearly half of the cardiovascular deaths occurring among individuals with a CAC score of zero, correctly reclassifying them into high- or very high–risk categories. This prognostic value was consistent across clinically relevant subgroups, including younger individuals in whom CAC may underestimate risk. This study presents and validates a novel algorithm that allows measurement of FAI score from noncontrast CT. Because CAC scoring is performed on noncontrast cardiac CT, coronary inflammation can now be quantified from images already acquired as part of routine cardiovascular risk assessment, without additional imaging, radiation exposure, contrast administration, or scan time. This could potentially extend coronary inflammation assessment to primary prevention and in patients who are intolerant to iodinated contrast agents, coming as a practical adjunct to routine CAC scoring.

At the opposite end of the atherosclerotic spectrum, coronary calcification has been proposed as a marker of potential plaque stabilization and reduced inflammatory activity.^26^ In support of this notion, densely calcified plaques (eg, “1K” Hounsfield unit plaques on cardiac CT) have previously been associated with lower plaque vulnerability and a reduced risk of acute coronary events.^15^ Although FAI score remained independently associated with cardiovascular risk in individuals with extensive coronary calcification, its relative prognostic contribution was attenuated. This may reflect a transition from biologically active, inflamed disease toward more heavily calcified, stable lesions, particularly in patients receiving preventive therapies (eg, statins).^13^ From a clinical perspective, coronary inflammation assessment may therefore provide the greatest incremental value in individuals with absent or minimal CAC, whose risk may be underestimated by CAC alone, and who typically receive limited preventive treatments. These findings may also inform patient selection for trials testing novel anti-inflammatory agents, particularly where systemic and coronary-specific measures of inflammation may be discordant. Specifically, populations with known advanced coronary calcification, such as those with chronic kidney disease enrolled in the recent ZEUS trial (NCT05021835), may prove resistant to anti-inflammatory treatments, likely due to discordance between systemic inflammation measured using high-sensitivity C-reactive protein and local coronary inflammation measured by FAI score.^29^

Importantly, residual inflammatory risk remains a major determinant of cardiovascular events despite intensive lipid-lowering therapy. Among statin-treated patients achieving low-density lipoprotein cholesterol (LDL-C) levels below 70 mg/dL, elevated systemic inflammation was strongly associated with cardiovascular events and mortality across the PROMINENT, REDUCE-IT, and STRENGTH trials.^30^ With low-dose colchicine now FDA-approved for cardiovascular risk reduction and additional targeted anti-inflammatory therapies under investigation,^31^ identifying patients with active coronary inflammation who may benefit from such therapies is increasingly important. NC FAI score may provide a scalable approach to identify this residual inflammatory risk from routinely acquired CAC scans, potentially enabling more individualized inflammation-targeted prevention. The use of the SAAS model to deliver this cloud-based technology can simplify access and ensure standardized analysis under a strict quality management system, offering the reproducibility and generalizability necessary for regulatory-cleared medical devices implemented in the clinic.

### Limitations

Our study has several limitations. First, the study population comprised symptomatic individuals with clinical indication for CCTA, potentially limiting generalizability to subclinical primary prevention populations, in whom NC FAI score requires further investigation. Second, NC FAI score was derived from noncontrast CAC scans commonly reconstructed at slice thickness of 2 to 3 mm, as compared to CCTA (typically 0.6-0.75 mm), resulting in lower spatial resolution and greater partial volume effects. Nevertheless, the ability to extract prognostically meaningful information on coronary inflammation from low–radiation dose scans highlights the potential to enhance cardiovascular risk assessment using routinely acquired images for CAC scoring, without additional image acquisition, radiation exposure, or scan time. Finally, the role of FAI score in selecting patients for targeted anti-inflammatory treatments in a clinical trial setting remains to be explored.

## Conclusions

Taken together, assessment of coronary inflammation from routine contrast CCTA or noncontrast CAC scans provides prognostic information beyond CAC scoring. The development and validation of NC FAI score extends this approach to routinely acquired noncontrast CAC imaging, identifying residual inflammatory risk among individuals with low or absent coronary calcification. Integration of coronary inflammation assessment into low-cost, low-radiation CAC imaging could refine preventive treatment decisions and expand inflammation-guided cardiovascular risk assessment to broader populations.

## Notes

Supplement 1. eAppendix. Supplemental Methods eFigure 1. Study Design eFigure 2. Development of Deep Learning Analysis for Non-Contrast CT eFigure 3. Prognostic Value of FAI Score in Individuals With No Known Atherosclerotic Cardiovascular Disease eFigure 4. Time-Dependent ROC Analysis for FAI Score Beyond Quantitative Plaque Volume in Predicting Cardiovascular Mortality eFigure 5. Clinical Decision Curve Analysis for Cardiovascular Mortality eFigure 6. Predictive Value of FAI Score for MACE in Different CAC Categories eFigure 7. Cardiovascular Risk Stratification by FAI Score in Individuals With No Coronary Calcification or No Visible Coronary Plaque eFigure 8. Continuous Net Reclassification Analysis eTable 1. ICD-10 Definitions of Clinical Events, Procedures, and Risk Factors eTable 2. Technical CT/CCTA Characteristics of the Derivation Cohort for the Segmentation Algorithm eTable 3. Predictive Value of FAI ScoreCCTA and FAI ScoreNC per Coronary Artery, for Cardiovascular Mortality and MACE eTable 4. Predictive Value of FAI ScoreCCTA and FAI ScoreNC per Coronary Artery, for Myocardial Infarction, New Heart Failure eTable 5. Competing Risk Analysis for Cardiovascular Mortality eTable 6. Cardiovascular Mortality Events per Risk Category eReferences.

Supplement 2. ORFAN Consortium

Supplement 3. Data Sharing Statement

## References

[hoi260066r1] Golub IS, Termeie OG, Kristo S, . Major global coronary artery calcium guidelines. JACC Cardiovasc Imaging. 2023;16(1):98-117. doi:10.1016/j.jcmg.2022.06.018 36599573

[hoi260066r2] Mitchell JD, Paisley R, Moon P, Novak E, Villines TC. Coronary artery calcium and long-term risk of death, myocardial infarction, and stroke: the Walter Reed Cohort Study. JACC Cardiovasc Imaging. 2018;11(12):1799-1806. doi:10.1016/j.jcmg.2017.09.003 29153576

[hoi260066r3] Agatston AS, Janowitz WR, Hildner FJ, Zusmer NR, Viamonte M Jr, Detrano R. Quantification of coronary artery calcium using ultrafast computed tomography. J Am Coll Cardiol. 1990;15(4):827-832. doi:10.1016/0735-1097(90)90282-T 2407762

[hoi260066r4] Budoff MJ, Young R, Burke G, . Ten-year association of coronary artery calcium with atherosclerotic cardiovascular disease (ASCVD) events: the Multi-Ethnic Study of Atherosclerosis (MESA). Eur Heart J. 2018;39(25):2401-2408. doi:10.1093/eurheartj/ehy217 29688297 PMC6030975

[hoi260066r5] Winther S, Schmidt SE, Mayrhofer T, . Incorporating coronary calcification into pre-test assessment of the likelihood of coronary artery disease. J Am Coll Cardiol. 2020;76(21):2421-2432. doi:10.1016/j.jacc.2020.09.585 33213720

[6] Vrints C, Andreotti F, Koskinas KC, ; ESC Scientific Document Group. 2024 ESC guidelines for the management of chronic coronary syndromes. Eur Heart J. 2024;45(36):3415-3537. doi:10.1093/eurheartj/ehae177 39210710

[7] Winther S, Schmidt SE, Foldyna B, . Coronary calcium scoring improves risk prediction in patients with suspected obstructive coronary artery disease. J Am Coll Cardiol. 2022;80(21):1965-1977. doi:10.1016/j.jacc.2022.08.805 PMC10466200 36396197

[hoi260066r8] Agha AM, Pacor J, Grandhi GR, . The prognostic value of CAC zero among individuals presenting with chest pain: a meta-analysis. JACC Cardiovasc Imaging. 2022;15(10):1745-1757. doi:10.1016/j.jcmg.2022.03.031 36202453

[hoi260066r9] Sarwar A, Shaw LJ, Shapiro MD, . Diagnostic and prognostic value of absence of coronary artery calcification. JACC Cardiovasc Imaging. 2009;2(6):675-688. doi:10.1016/j.jcmg.2008.12.031 19520336

[10] Grundy SM, Stone NJ, Bailey AL, . 2018 AHA/ACC/AACVPR/AAPA/ABC/ACPM/ADA/AGS/APhA/ASPC/NLA/PCNA guideline on the management of blood cholesterol: executive summary: a report of the American College of Cardiology/American Heart Association Task Force on Clinical Practice Guidelines. J Am Coll Cardiol. 2019;73(24):3168-3209. doi:10.1016/j.jacc.2018.11.002 30423391

[hoi260066r11] Blumenthal RS, Morris PB, Gaudino M, . 2026 ACC/AHA/AACVPR/ABC/ACPM/ADA/AGS/APhA/ASPC/NLA/PCNA guideline on the management of dyslipidemia: a report of the American College of Cardiology/American Heart Association Joint Committee on Clinical Practice Guidelines. J Am Coll Cardiol. 2026;87(19):2624-2757. doi:10.1016/j.jacc.2025.11.016 41824590

[hoi260066r12] Chan K, Wahome E, Tsiachristas A, ; ORFAN Consortium. Inflammatory risk and cardiovascular events in patients without obstructive coronary artery disease: the ORFAN multicentre, longitudinal cohort study. Lancet. 2024;403(10444):2606-2618. doi:10.1016/S0140-6736(24)00596-8 38823406 PMC11664027

[hoi260066r13] Antoniades C, Chan K. Calcification vs inflammation: the modern toolkit for cardiovascular risk assessment. JACC Cardiovasc Imaging. 2024;17(10):1225-1228. doi:10.1016/j.jcmg.2024.08.006 39384268

[hoi260066r14] Criqui MH, Denenberg JO, Ix JH, . Calcium density of coronary artery plaque and risk of incident cardiovascular events. JAMA. 2014;311(3):271-278. doi:10.1001/jama.2013.282535 24247483 PMC4091626

[hoi260066r15] van Rosendael AR, Narula J, Lin FY, . Association of high-density calcified 1K plaque with risk of acute coronary syndrome. JAMA Cardiol. 2020;5(3):282-290. doi:10.1001/jamacardio.2019.5315 31968065 PMC6990946

[hoi260066r16] Miedema MD, Dardari ZA, Nasir K, . Association of coronary artery calcium with long-term, cause-specific mortality among young adults. JAMA Netw Open. 2019;2(7):e197440. doi:10.1001/jamanetworkopen.2019.7440 31322693 PMC6646982

[hoi260066r17] Libby P, Ridker PM, Hansson GK; Leducq Transatlantic Network on Atherothrombosis. Inflammation in atherosclerosis: from pathophysiology to practice. J Am Coll Cardiol. 2009;54(23):2129-2138. doi:10.1016/j.jacc.2009.09.009 19942084 PMC2834169

[hoi260066r18] Oikonomou EK, Marwan M, Desai MY, . Non-invasive detection of coronary inflammation using computed tomography and prediction of residual cardiovascular risk (the CRISP CT study): a post-hoc analysis of prospective outcome data. Lancet. 2018;392(10151):929-939. doi:10.1016/S0140-6736(18)31114-0 30170852 PMC6137540

[hoi260066r19] Antonopoulos AS, Sanna F, Sabharwal N, . Detecting human coronary inflammation by imaging perivascular fat. Sci Transl Med. 2017;9(398):eaal2658. doi:10.1126/scitranslmed.aal2658 28701474

[20] Oikonomou EK, Antonopoulos AS, Schottlander D, . Standardized measurement of coronary inflammation using cardiovascular computed tomography: integration in clinical care as a prognostic medical device. Cardiovasc Res. 2021;117(13):2677-2690. doi:10.1093/cvr/cvab286 34450625

[hoi260066r21] Oikonomou EK, Williams MC, Kotanidis CP, . A novel machine learning-derived radiotranscriptomic signature of perivascular fat improves cardiac risk prediction using coronary CT angiography. Eur Heart J. 2019;40(43):3529-3543. doi:10.1093/eurheartj/ehz592 31504423 PMC6855141

[hoi260066r22] Mensah GA, Arnold N, Prabhu SD, Ridker PM, Welty FK. Inflammation and cardiovascular disease: 2025 ACC scientific statement: a report of the American College of Cardiology. J Am Coll Cardiol. 2026;87(11):1381-1404. doi:10.1016/j.jacc.2025.08.047 41020749

[23] Detrano R, Guerci AD, Carr JJ, . Coronary calcium as a predictor of coronary events in four racial or ethnic groups. N Engl J Med. 2008;358(13):1336-1345. doi:10.1056/NEJMoa072100 18367736

[24] Erbel R, Möhlenkamp S, Moebus S, ; Heinz Nixdorf Recall Study Investigative Group. Coronary risk stratification, discrimination, and reclassification improvement based on quantification of subclinical coronary atherosclerosis: the Heinz Nixdorf Recall study. J Am Coll Cardiol. 2010;56(17):1397-1406. doi:10.1016/j.jacc.2010.06.030 20946997

[hoi260066r25] Hussain A, Ballantyne CM, Nambi V. Zero coronary artery calcium score: desirable, but enough? Circulation. 2020;142(10):917-919. doi:10.1161/CIRCULATIONAHA.119.045026 32897743

[hoi260066r26] Blankstein R, Shaw LJ, Chandrashekhar Y. Coronary calcium in the last decade: where are we now. JACC Cardiovasc Imaging. 2025;18(3):399-401. doi:10.1016/j.jcmg.2025.02.001 40044328

[27] Fujimoto D, Kinoshita D, Suzuki K, . Relationship between calcified plaque burden, vascular inflammation, and plaque vulnerability in patients with coronary atherosclerosis. JACC Cardiovasc Imaging. 2024;17(10):1214-1224. doi:10.1016/j.jcmg.2024.07.013 39243232

[28] Yong Y, Giovannucci J, Pang SN, . Coronary artery calcium density and risk of cardiovascular events: a systematic review and meta-analysis. JACC Cardiovasc Imaging. 2025;18(3):294-304. doi:10.1016/j.jcmg.2024.07.024 39243235

[hoi260066r29] Ridker PM, Baeres FMM, Hveplund A, . Rationale, design, and baseline clinical characteristics of the Ziltivekimab Cardiovascular Outcomes Trial: interleukin-6 inhibition and atherosclerotic event rate reduction. JAMA Cardiol. 2026;11(1):89-97. doi:10.1001/jamacardio.2025.4491 41369941

[hoi260066r30] Ridker PM, Lei L, Louie MJ, ; CLEAR Outcomes Investigators. Inflammation and cholesterol as predictors of cardiovascular events among 13 970 contemporary high-risk patients with statin intolerance. Circulation. 2024;149(1):28-35. doi:10.1161/CIRCULATIONAHA.123.066213 37929602 PMC10752259

[hoi260066r31] Potere N, Bonaventura A, Abbate A. Novel therapeutics and upcoming clinical trials targeting inflammation in cardiovascular diseases. Arterioscler Thromb Vasc Biol. 2024;44(12):2371-2395. doi:10.1161/ATVBAHA.124.319980 39387118 PMC11602387

