# Supplementary material for: Assessment of Coronary Inflammatory Risk From Noncontrast Cardiac Computed Tomography
Source: JAMA Cardiol. 2026 Nov 11:e265006. Online ahead of print. doi: 10.1001/jamacardio.2026.5006 (PMC13756199; doi:10.1001/jamacardio.2026.5006)
Supplement: Supplementary file 1 [file jamacardiol-e265006-s001.pdf]

Supplemental Online Content

Chan K, Gissler MC, Sobirov I, et al; ORFAN Consortium. Assessment of coronary inflammatory risk from noncontrast cardiac computed tomography. *JAMA Cardiol*. Published online November 11, 2026. doi:10.1001/jamacardio.2026.5006

eAppendix. Supplemental Methods

eFigure 1. Study Design

eFigure 2. Development of Deep Learning Analysis for Non-Contrast CT

eFigure 3. Prognostic Value of FAI Score in Individuals With No Known Atherosclerotic Cardiovascular Disease

eFigure 4. Time-Dependent ROC Analysis for FAI Score Beyond Quantitative Plaque Volume in Predicting Cardiovascular Mortality

eFigure 5. Clinical Decision Curve Analysis for Cardiovascular Mortality

eFigure 6. Predictive Value of FAI Score for MACE in Different CAC Categories

eFigure 7. Cardiovascular Risk Stratification by FAI Score in Individuals With No Coronary Calcification or No Visible Coronary Plaque

eFigure 8. Continuous Net Reclassification Analysis

eTable 1. *ICD-10* Definitions of Clinical Events, Procedures, and Risk Factors

eTable 2. Technical CT/CCTA Characteristics of the Derivation Cohort for the Segmentation Algorithm

eTable 3. Predictive Value of FAI Score$_{CCTA}$ and FAI Score$_{NC}$ per Coronary Artery, for Cardiovascular Mortality and MACE

eTable 4. Predictive Value of FAI Score$_{CCTA}$ and FAI Score$_{NC}$ per Coronary Artery, for Myocardial Infarction, New Heart Failure

eTable 5. Competing Risk Analysis for Cardiovascular Mortality

eTable 6. Cardiovascular Mortality Events per Risk Category

eReferences.

This supplemental material has been provided by the authors to give readers additional information about their work.

© 2026 Chan K et al; ORFAN Consortium. *JAMA Cardiology*.

eAppendix. Supplemental Methods

1.1 Collection of outcomes data and patient characteristics

The ORFAN study was approved by the Oxfordshire Research Ethics Committee (REC 15/SC/0545) and the UK Confidentiality Advisory Group (20/CAG/0157). This is an academic programme funded by the British Heart Foundation and the UK Government via the National Institute of Health Research (NIHR), while it is sponsored and designed by the University of Oxford. The study is overseen by a Steering Committee with representatives of the key geographical regions contributing into the study, that sets the publications and data sharing strategy. Demographic patient characteristics and data on clinical outcomes in the Oxford Risk Factors and Non-invasive imaging (ORFAN) study were retrieved through data-linkage with the NHS England (NHS Digital) and National Institute for Cardiovascular Outcomes Research (NICOR) datasets, as described previously[1]. This includes Myocardial Infarction National Audit Project (MINAP) and National Adult Cardiac Surgery Audit (NACSA), the National Audit of Percutaneous Coronary Interventions (NAPCI), Hospital Episode Statistics, HES and Office for National Statistics (ONS) for nationally registered mortality from 2005-2024. Local clinical databases were interrogated by the respective ORFAN investigators to obtain data on demographics, cardiovascular risk factors, and medication records using local electronic health records, the radiographic safety questionnaires, the clinical CCTA reports and any other locally available resources. Patient demographics, cardiovascular risk factors and clinical outcomes, including major adverse cardiac events (MACE), defined as acute myocardial infarction, new heart failure, and cardiovascular mortality, were also obtained from NHS England datasets using ICD-10 codes. Risk factors were further cross-referenced with the local hospital's electronic patient records (including the GP referral letters from the General Practitioners) as well as the NHS's Pharmacy Prescriptions databases where all medications prescribed to a patient are centrally recorded. New heart failure was defined as the first occurrence of a primary diagnosis of heart failure occurring after the index CCTA in patients

© 2026 Chan K et al; ORFAN Consortium. *JAMA Cardiology*.

with no prior history of heart failure. Cardiovascular causes for mortality were defined as all unified causes of death under the ICD-10 diagnostic code I.

Adjudication of clinical events was performed centrally by the ORFAN events adjudication committee that was blinded to the CCTA analyses. The ORFAN events adjudication team cross-referenced information from NHS England, with the events recorded in the NICOR / MINAP datasets, as defined in the study documentation. When disagreements between the various sources were identified, the events adjudication team reviewed local hospital records as well as other sources of information, such as revascularisation procedures at the time of the reported event (NICOR individual procedures datasets), and further reviewed with a senior referee to reach consensus. A similar approach was followed by the central adjudication team to resolve discrepancies in any other clinical information between the different data sources. Coronary revascularisation procedures (Percutaneous coronary intervention (PCI), coronary artery bypass grafting) in relation to the index cardiac event were extracted using the Classification of Interventions and Procedures (OPCS-4) codes and cross-validated with the National Adult Cardiac Surgery Audit (NACSA) adult cardiac surgery and the National Audit of Percutaneous Coronary Interventions (NAPCI) databases. All analyses were blinded to the outcome data, clinical CCTA reports and local databases.

1.2 Cardiac computed tomography technical parameters and FAI Score analysis

Participants in the ORFAN study in the UK underwent CCTA scans at NHS hospitals, including Oxford University Hospitals, Royal Brompton and Harefield Hospitals, Royal United Hospital Bath, Royal Papworth Hospital, Guys and St Thomas Hospital, Leicester University Hospital, Milton Keynes Hospital, Newcastle Hospitals, Liverpool Heart and Chest Hospitals, University Leeds Teaching Hospitals and Barts Health. All scans were performed according to local clinical procedures, with minimal variance between sites. Heart rate was optimised with intravenous beta-blockers, and sublingual glyceryl trinitrate was administered as needed to achieve coronary vasodilatation. CCTA was performed following intravenous injection of 50-80 mL of iodine-based contrast medium (typically Niopam 370, BRACCO UK Ltd) at a flow

© 2026 Chan K et al; ORFAN Consortium. *JAMA Cardiology*.

rate of 5.5-6.5 mL/sec (axial slice thickness on Canon scanners was typically 0.5 mm, with Siemens typically between 0.65 mm and 0.75 mm, rotation time was 0.35 seconds, detector coverage was up to 160 mm, and tube energy was selected based on body habitus and according to local clinical practice). Prospective image acquisition was performed by ECG-gating at 75% of the cardiac cycle (with 100 msec padding if required). If not possible, retrospective image acquisition was used (e.g., in the presence of irregular rhythm). Vendors of CCTA scanners included: 320-slice scanner (Aquilion ONE, Canon Medical Systems); 2 × 128-slice Definition Flash scanner (Siemens Healthcare, Erlangen, Germany); 2 × 128-slice Somatom Drive (Siemens Healthcare, Munich, Germany); 2 × 192-slice Somatom Force CT scanner (Siemens Healthcare, Forchheim, Germany), 256-slice Philips Brillance iCT, 256-slice General Electric Medical systems Revolution and 64-slice General Electric LightSpeed Ultra (General Electric Healthcare, Chicago, USA). For all ORFAN sites, scans with profoundly poor-quality images, more than 2 missing slices, severe beam hardening or significant motion artefacts were excluded from the analysis. Minor to moderate scan artefacts were acceptable and included in the analysis. FAI Score was measured in the proximal segments of the three coronary arteries as previously described[2] using an unsupervised deep learning model developed within the CaRi-Heart toolkit.

1.3 Development and validation of FAI Score$_{NC}$

In the derivation cohort (n = 6818), contrast-enhanced CCTA and non-contrast CT (NCCT) scans were co-registered to enable accurate transfer of perivascular adipose tissue (PVAT) annotations. A generative adversarial network (GAN) was trained to generate synthetic contrast-enhanced scans (sCT) from NCCT, improving delineation of the vessel wall-PVAT interface and facilitating segmentation. In particular, the sCT was first used to evaluate the quality of the transferred mask on NCCT.

NCCT scans with high-quality PVAT masks were used to train and validate a dedicated convolutional neural network (CNN)-based deep learning model for segmenting PVAT regions. We refined the model through an iterative learning process. In the first iteration, the model was

trained and validated using 2571 cases (80:20 split) to establish a baseline. The baseline model achieved interclass correlation coefficients (ICC) of 0.903 (95% CI: [0.90-0.91]), 0.846 (95% CI: [0.83-0.86]), and 0.904 (95% CI: [0.90-0.91]) for RCA, LCX, and LAD, respectively. During the iterative learning process, the model's outputs were manually reviewed by expert clinical readers and, when needed, corrected using sCT as a guide. After three iterations, the entire derivation cohort, after excluding cases with technical issues (n = 49), was used to train and validate the model in an 80:20 split. The performance of the final model improved to ICCs of 0.903 (95% CI: [0.90-0.91]), 0.846 (95% CI: [0.83-0.86]), and 0.904 (95% CI: [0.90-0.91]) for RCA, LCX, and LAD, respectively.

The deep learning model architecture was previously described.[3] Briefly, the model employs a conventional 3D U-shaped architecture with residual connections and squeeze-and-excitation normalization blocks. Four encoder blocks progressively downsample the data, while four corresponding decoder blocks reconstruct the image to its original resolution. Skip connections between corresponding encoder and decoder blocks enable information flow across the network. Adapted for PVAT segmentation, the scans were aligned to a uniform direction and orientation, resampled to isotropic spacing of 1x1x1 mm^3, clipped to the [-1024, 1024] Hounsfield unit range, and normalized to [0, 1] before being fed into the model. The model was trained for 100 epochs using an AdamW optimizer with a learning rate of 1e-3, a cosine annealing scheduler with T_0 set to 25 and a minimum η of 1e-5, and an equally weighted sum of Dice and Cross-Entropy losses. Training was performed on a single Tesla V100 GPU using Python version 3.9.18 and PyTorch version 2.1.2.

Finally, the model was deployed on the external validation cohort (n = 19176). An automatic quality control method encoded in the model flagged 5.4% of these cases for manual review, and of those, 66 cases (0.34% of the whole cohort) were rejected due to poor scan quality (see **eFigure 1** for detailed list of justification for scan rejection).

Following segmentation, the CaRi-Heart toolkit was used to calculate FAI $Score_{NC}$ using the same proprietary algorithm applied for CCTAs, that performs corrections for several technical

© 2026 Chan K et al; ORFAN Consortium. *JAMA Cardiology*.

scan parameters as well as local anatomical and biological factors, enabling the generation of individual FAI $Score_{NC}$ for each coronary artery. In the independent external validation cohort, FAI $Score_{NC}$ demonstrated a strong correlation with the reference CCTA-derived FAI $Score_{CCTA}$ across all three major coronary arteries (r = 0.80–0.86, all P < 0.001). Bland–Altman analysis further demonstrated excellent agreement between FAI $Score_{NC}$ and FAI $Score_{CCTA}$, with minimal systematic bias and narrow limits of agreement, supporting the accuracy and robustness of the non-contrast FAI Score (**eFigure 2**).

© 2026 Chan K et al; ORFAN Consortium. *JAMA Cardiology*.

eFigure 1. Study Design

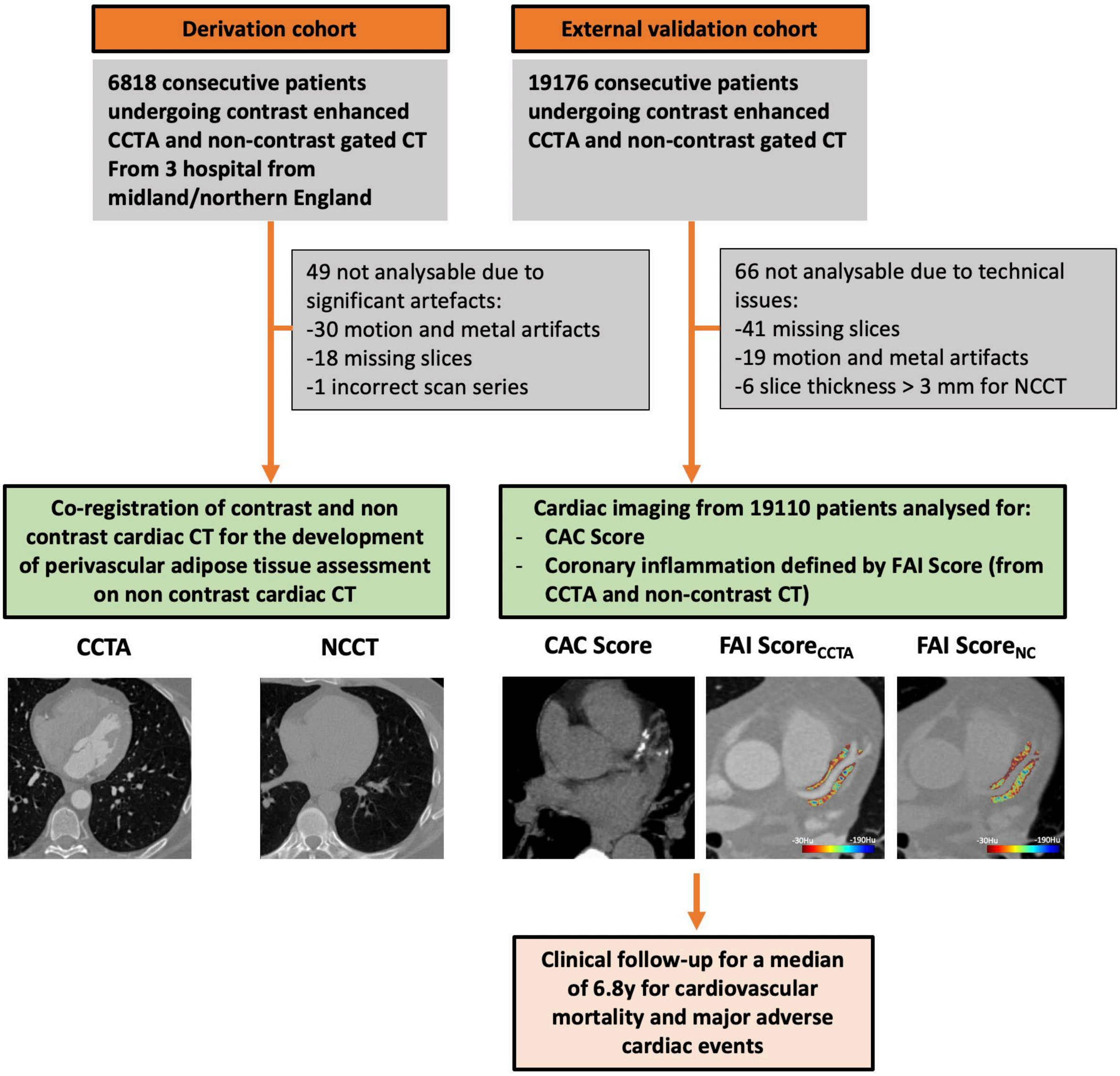

Overview of study design. Abbreviations: CAC, Coronary artery calcium; CCTA, Coronary Computed Tomography; FAI Score, Fat attenuation Index Score; NCCT, Non-contrast gated Computed Tomography

© 2026 Chan K et al; ORFAN Consortium. *JAMA Cardiology*.

eFigure 2. Development of Deep Learning Analysis for Non-Contrast CT

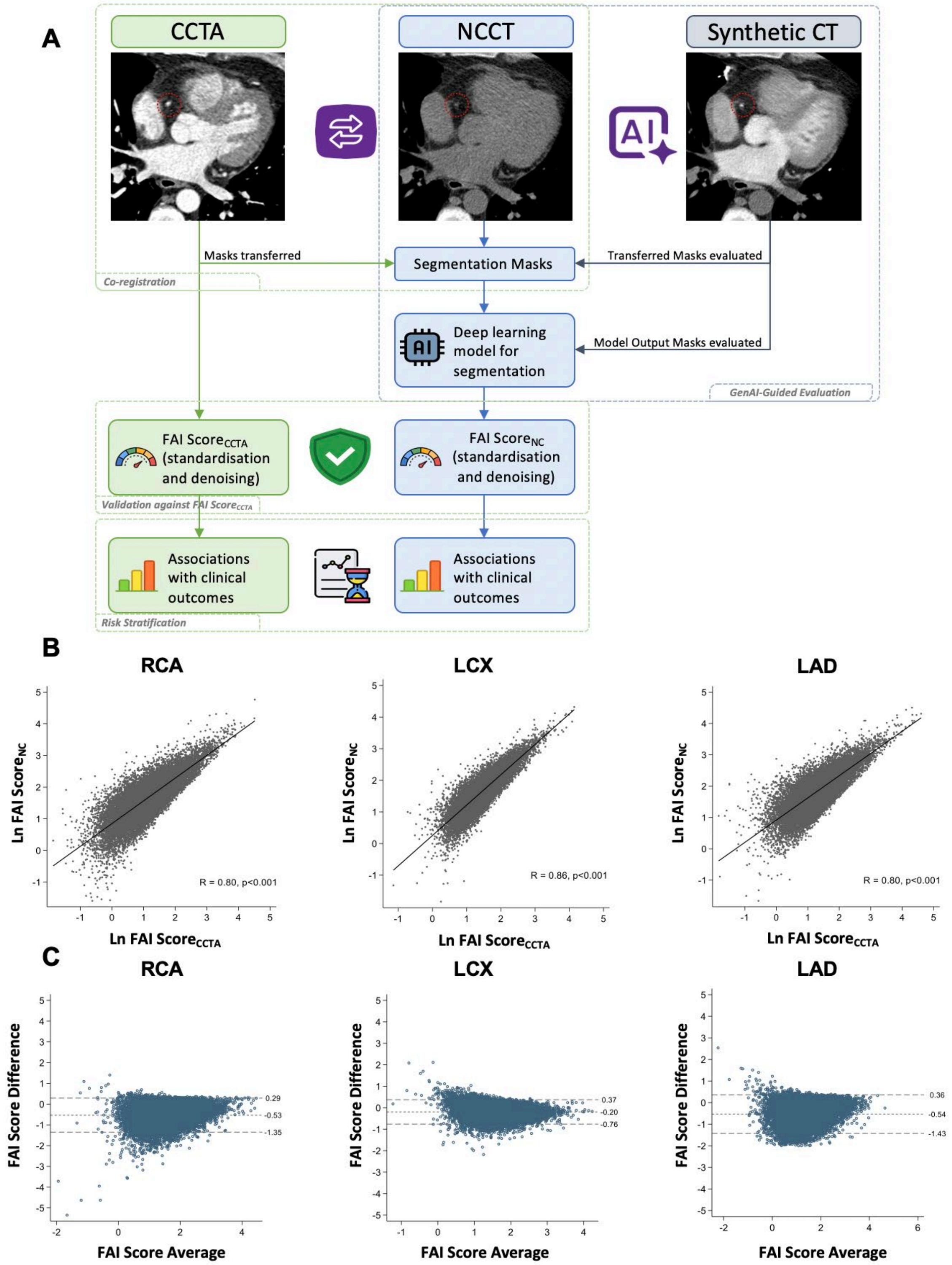

Development and evaluation of FAI Score$_{NC}$ from non-contrast computed tomography (A). FAI Score$_{CCTA}$ and FAI Score$_{NC}$ were strongly correlated (r> 0.8) across all three coronary arteries (B). Bland-Altman plots showed good agreement between FAI Score$_{CCTA}$ and FAI Score$_{NC;}$ Dashed lines represent the limits of agreements from -1.96 to +1.96 standard deviation (C). Abbreviations: CCTA, coronary computed tomography angiography; FAI, Fat attenuation index; LAD, left anterior descending coronary artery; LCX, left circumflex artery; RCA, right coronary artery; NCCT, non-contrast computed tomography.

© 2026 Chan K et al; ORFAN Consortium. *JAMA Cardiology*.

eFigure 3. Prognostic Value of FAI Score in Individuals With No Known Atherosclerotic Cardiovascular Disease

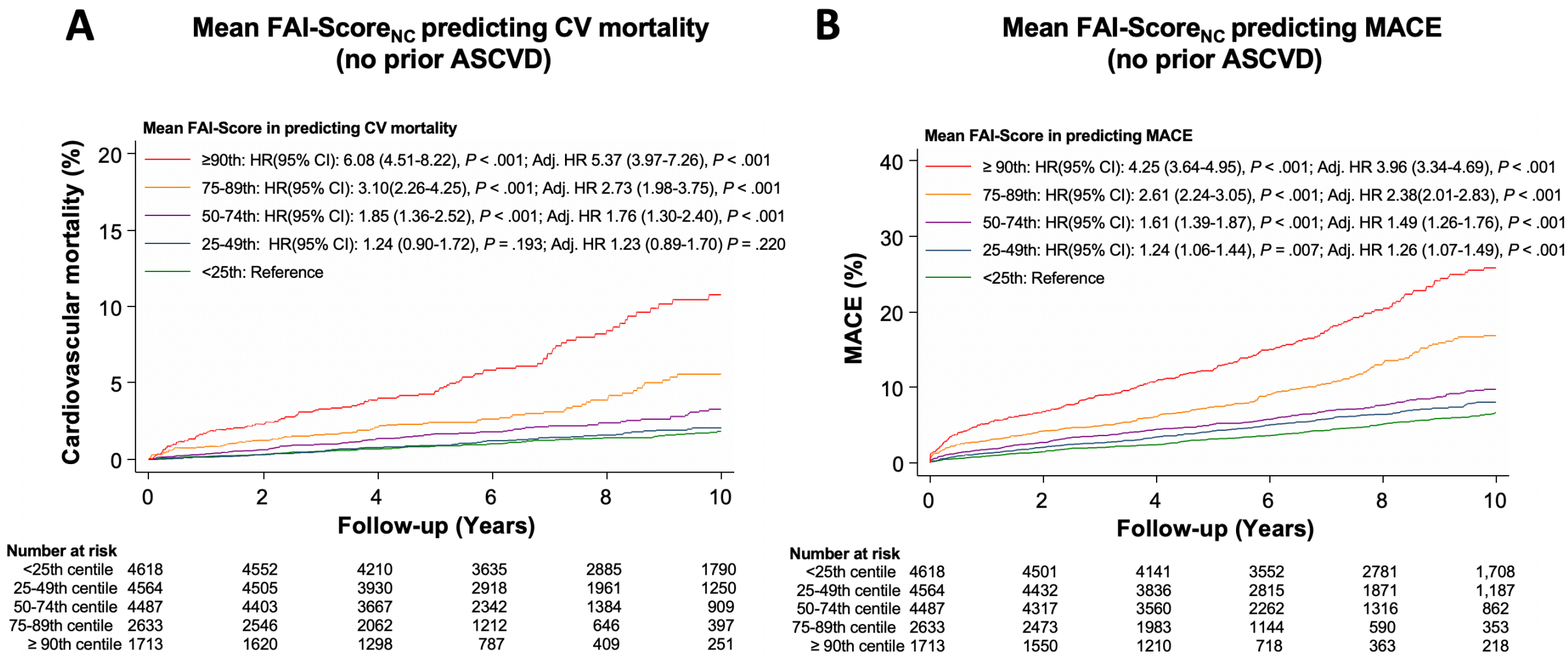

Kaplan–Meier curves for the prognostic value of mean FAI Score$_{NC}$ for cardiovascular mortality (A) and MACE (B) in individuals with no known atherosclerotic cardiovascular disease (ASCVD) prior to CT imaging, defined as no history of myocardial infarction, stroke, or coronary revascularization. Adj. HR represent HRs after adjustment for CAC Score. Abbreviations: CV, cardiovascular; FAI Score$_{NC}$, Fat attenuation index from non-contrast CT; HR, hazard ratio; SD, standard deviation.

© 2026 Chan K et al; ORFAN Consortium. *JAMA Cardiology*.

eFigure 4. Time-Dependent ROC Analysis for FAI Score Beyond Quantitative Plaque Volume in Predicting Cardiovascular Mortality

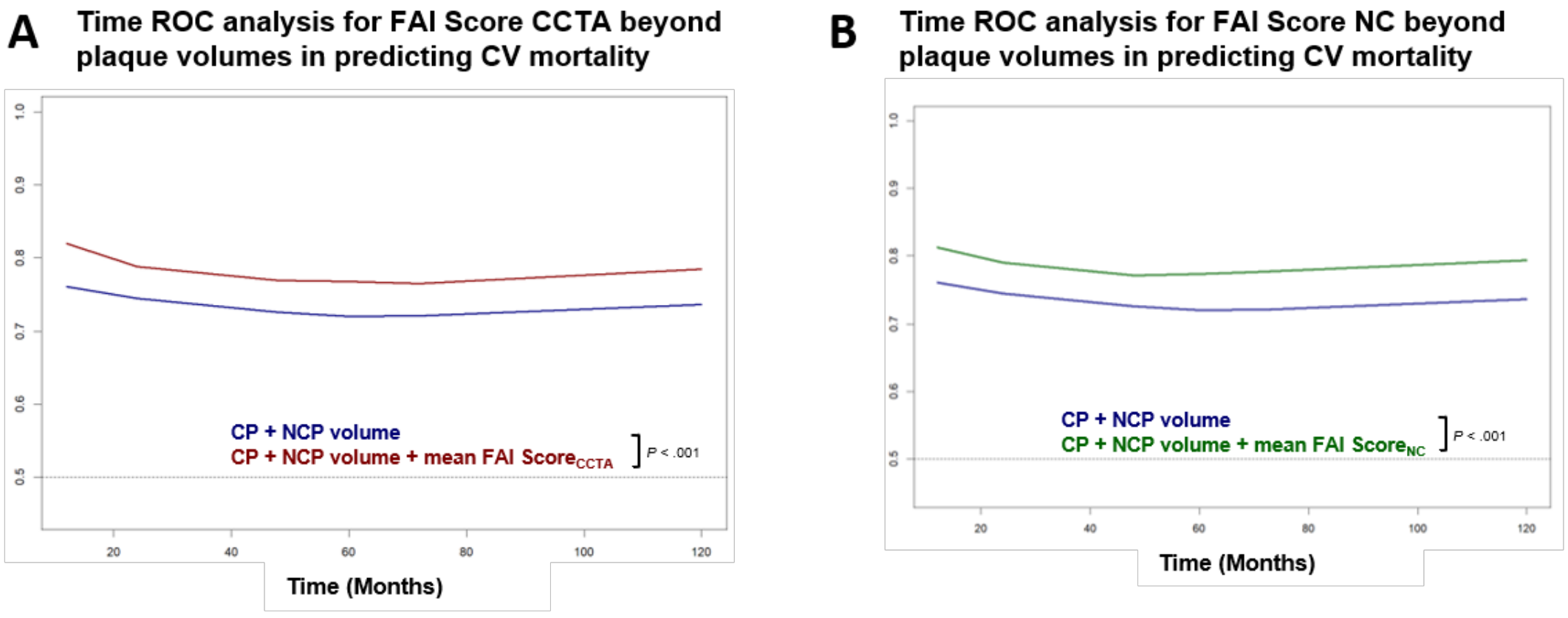

Abbreviations: CP, calcified plaque; FAI, fat attenuation index ; NCP, non calcified plaque ; ROC, receiver operating curve

© 2026 Chan K et al; ORFAN Consortium. *JAMA Cardiology*.

eFigure 5. Clinical Decision Curve Analysis for Cardiovascular Mortality

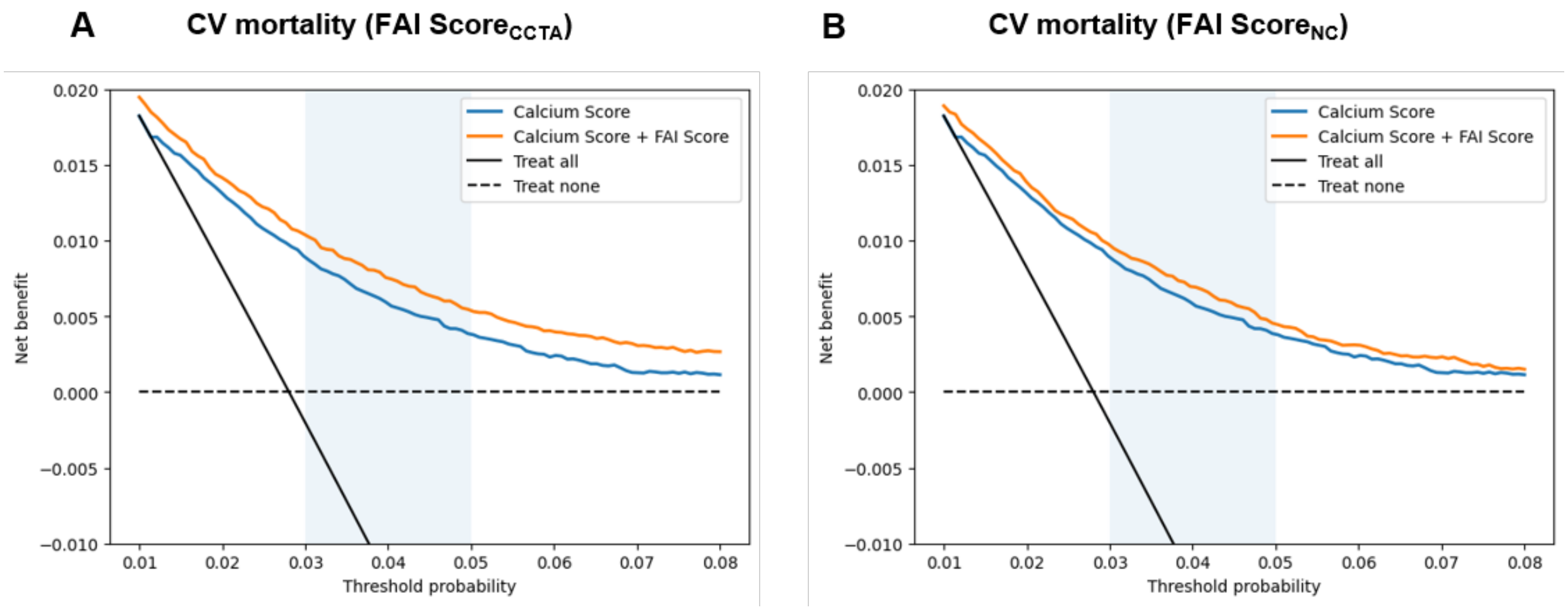

Decision curve analysis for cardiovascular mortality using coronary calcium score as base model and the addition of mean FAI Score$_{CCTA}$ (A) FAI Score$_{NC}$ (B) over a 10year horizon. The horizontal reference line (dotted) represents the strategy of treating no patients, whereas the continuous black line represents the strategy of treating all patients. Across the clinically relevant 10-year risk thresholds (approximately 3%-5%), the model that incorporates FAI Score$_{CCTA}$ or FAI Score$_{NC}$ in addition to coronary calcium score demonstrates consistently greater net benefit compared with the baseline model, supporting improved clinical utility. Abbreviations: CV, cardiovascular; FAI Score, fat attenuation index Score.

© 2026 Chan K et al; ORFAN Consortium. *JAMA Cardiology*.

eFigure 6. Predictive Value of FAI Score for MACE in Different CAC Categories

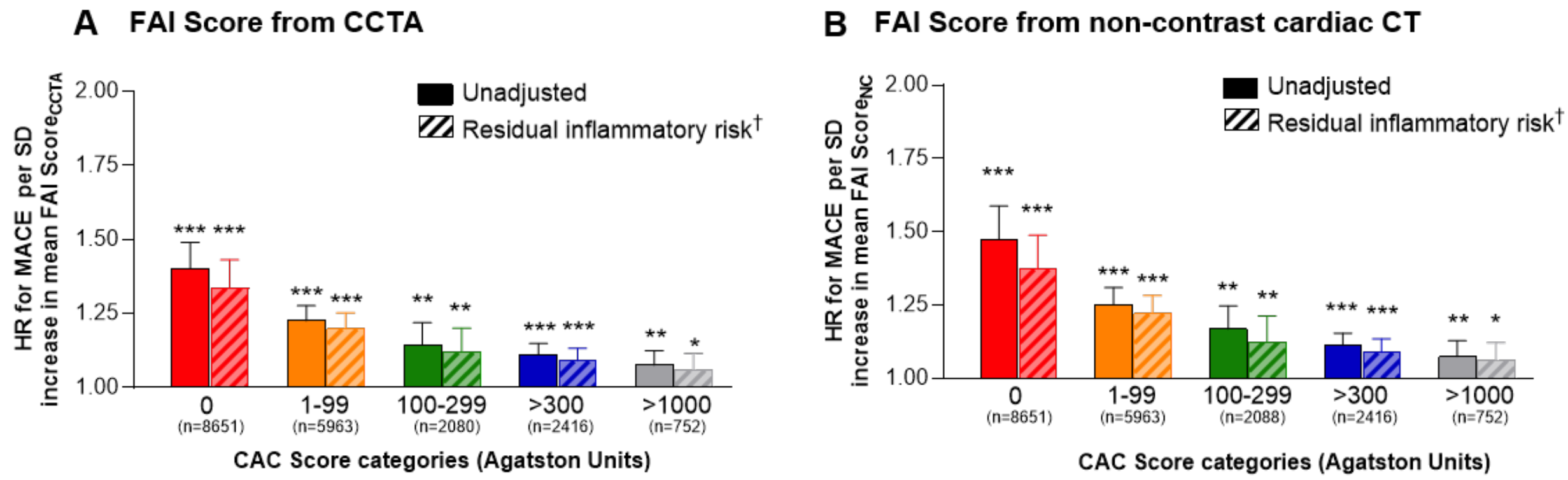

Coronary inflammation quantified by mean FAI Score$_{CCTA}$ (A) FAI Score$_{NC}$ (B) as a predictor of MACE in patients with different levels of coronary artery calcification defined by CAC Score categories. Error bars indicate 95% confidence intervals. †HR per SD after adjustment for age, sex, hypertension, hyperlipidaemia, diabetes, smoking status and non-calcified plaque volume (mm3). Abbreviations: CAC, coronary artery calcification; FAI, fat attenuation index; MACE, major adverse cardiac events; HR, hazard ratio; SD, standard deviation. *p<0.05, **p<0.01, ***p<0.001

© 2026 Chan K et al; ORFAN Consortium. *JAMA Cardiology*.

eFigure 7. Cardiovascular Risk Stratification by FAI Score in Individuals With No Coronary Calcification or No Visible Coronary Plaque

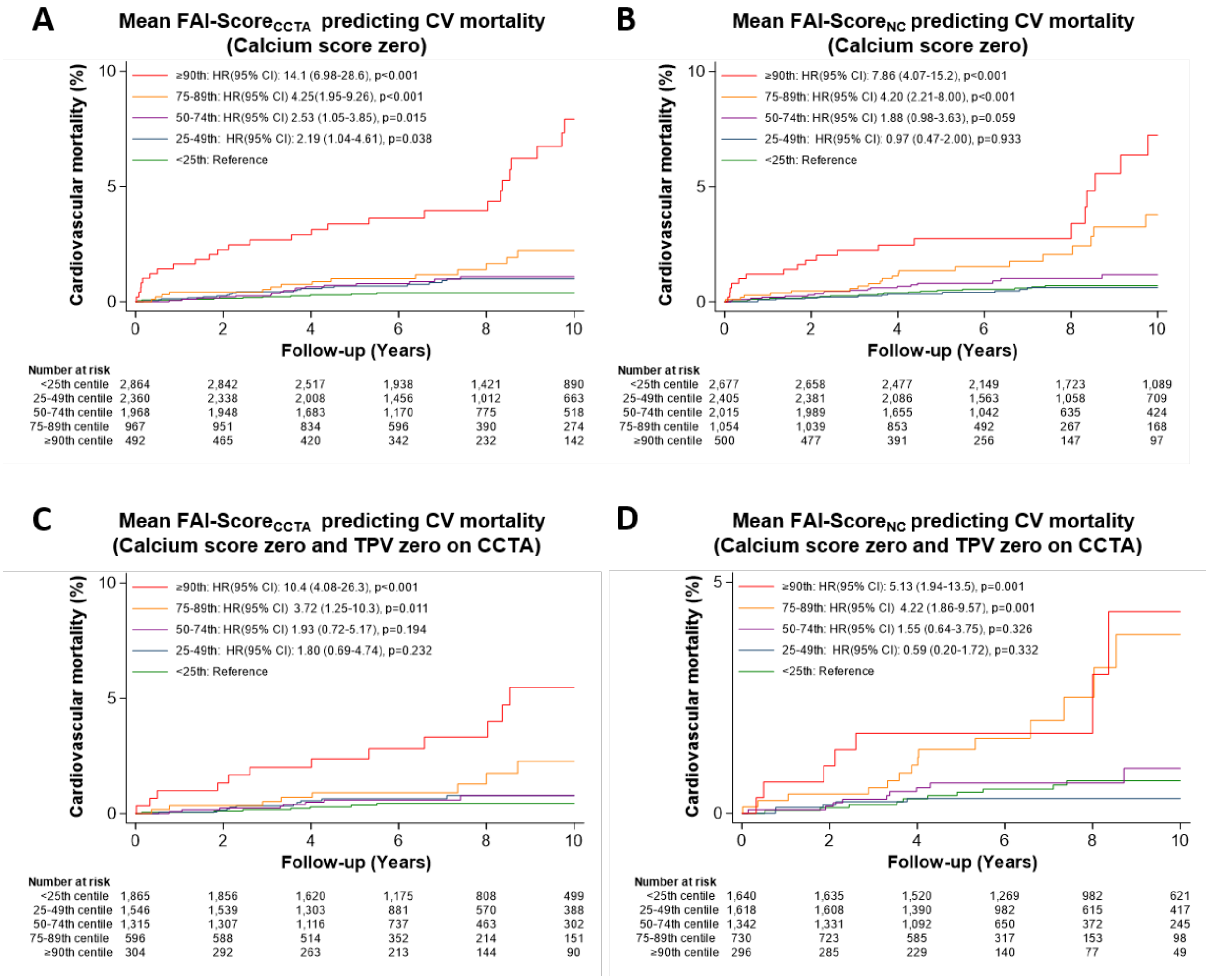

Kaplan–Meier curves showing cumulative cardiovascular mortality according to FAI Score percentile categories. Analyses are shown for individuals with a coronary artery calcium (CAC) score of zero and for individuals with no visible coronary plaque on CCTA (neither calcified nor non-calcified plaque), using FAI Score derived from contrast-enhanced CCTA and non-contrast CT, respectively. Hazard ratios (HRs) are shown relative to the <25th percentile group. TPV = quantitative total plaque volume (mm^3)

© 2026 Chan K et al; ORFAN Consortium. *JAMA Cardiology*.

eFigure 8. Continuous Net Reclassification Analysis

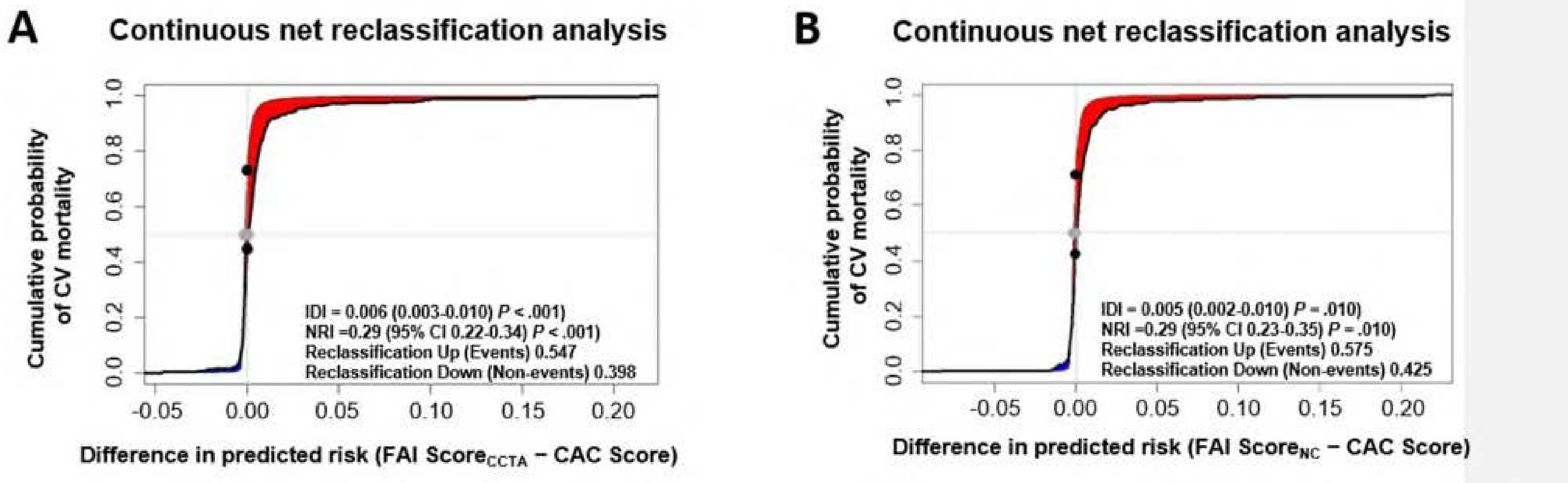

© 2026 Chan K et al; ORFAN Consortium. *JAMA Cardiology*.

eTable 1. *ICD-10* Definitions of Clinical Events, Procedures, and Risk Factors

		ICD-10 Codes	OPCS-4 Codes
**Outcomes**			
	Myocardial infarction	STEMI [I21.0,I21.1, I21.2, I21.3, I22.0, I22.1, I22.8], NSTEMI [I21.4, I21.9, I22.9] and unspecified MI [I23.x, I24.1, I24.8, I24.9]	
	Heart failure	I11.0, I13.0, I13.2, I25.5, I42.0, I42.5, I42.8, I42.9, I50.0, I50.1, I50.9, K76.1	
	Ischemic stroke	I63.x	
	Cardiac mortality	I11, I13, I20, I21, I22, I23, I24, I25, I30, I31, I32, I33, I34, I35, I36, I37, I38, I39, I40, I41, I42, I43, I44, I45, I46, I47, I48, I49, I50, I51, I52	
	Cardiovascular mortality	I.x	
	MI mortality	I21.0,I21.1, I21.2, I21.3, I22.0, I22.1, I22.8, I21.4, I21.9, I22.9, I23.x, I24.1, I24.8, I24.9	
**Procedures**			
	PCI		K49.x-50.x, K75.x
	CABG		K4[0123456] [123489], K45[56], K4[6]5
**Risk factors**			
	Hypertension	I10.x, I11.x, I12.x, I13.x, I15.x	
	Hyperlipidaemia	E78.0, E78.1, E78.2, E78.4, E78.5 (excluding E78.3)	
	Diabetes mellitus type 1	E10.x	
	Diabetes mellitus type 2	E11.x	
	Diabetes mellitus other	E12.x, E13.x, E14.x	

ICD-10 International Classification of Disease 10th revision. Abbreviations: MI, myocardial infarction; NSTEMI, Non-ST-elevation myocardial infarction; STEMI, ST-elevation myocardial infarction.

© 2026 Chan K et al; ORFAN Consortium. *JAMA Cardiology*.

eTable 2. Technical CT/CCTA Characteristics of the Derivation Cohort for the Segmentation Algorithm

**Characteristics**	**N=6818**	**%**
Median age (IQR), years	61 (51-71)	
Male, n (%)	3275	48%
**Scanner type**		
Siemens SOMATOM FORCE	3337	49%
Siemens SOMATOM Definition Flash	3424	50%
Siemens SOMATOM go.Top	3	0%
Canon Aquilion	51	1%
GE Medical systems Revolution EVO	2	0.03%
**CCTA technical characteristics**		
Slice thickness		
0.50 mm	54	1%
0.60 mm	20	0.29%
0.75 mm	6740	99%
0.80 mm	2	0.03%
Tube voltage		
70 kVp	310	5%
80 kVp	904	13%
90 kVp	921	14%
100 kVp	2484	36%
110 kVp	288	4%
120 kVp	1875	28%
130 kVp	8	0.12%
135 kVp	3	0.04%
140 kVp	25	0.37%
Kernel Reconstruction	['B26f', 'B30f', 'CARDIAC', 'FC03', 'FC43', 'FC52', 'STANDARD', "['Br36d', '4']", "['Br40d', '2']", "['Br40d', '4']", "['Bv36d', '1']", "['Bv36d', '2']", "['Bv36d', '3']", "['Bv36f', '3']", "['Bv40d', '1']", "['Bv40d', '2']", "['Bv40d', '3']", "['Bv40d', '4']", "['Bv40d', '5']", "['Bv49d', '3']", "['Bv49d', '4']", "['I26f', '3']", "['I26f', '4']", "['I30f', '3']", "['I46f', '3']", "['I70f', '3']", "['Qr36d', '5']	
**NCCT technical characteristics**		
Slice thickness		
3.0 mm	4875	72%
2.5mm	2	0.03%
2.0mm	1940	28%
Tube voltage		
100 kVp	2069	30%
120 kVp	4748	70%
Kernel Reconstruction	['B35f', 'B36f', 'B60f', 'B70f', 'Bf36d', 'Bf40d', 'Bl57f', 'Br40f', 'FC12', 'STANDARD', 'Sa36d', 'Sa36f', "['Br36f', '3']", "['Br40f', '3']", "['Br59f', '3']", "['I30f', '3']", "['I36f', '3']", "['I70f', '3']", "['Qr36d', '3']", "['Qr36f', '3']", "['Qr59d', '3']", "['Qr59f', '3']	

© 2026 Chan K et al; ORFAN Consortium. *JAMA Cardiology*.

eTable 3. Predictive Value of FAI Score$_{CCTA}$ and FAI Score$_{NC}$ per Coronary Artery, for Cardiovascular Mortality and MACE

	Cardiovascular mortality				MACE			
	Total inflammatory risk		Residual inflammatory risk		Total inflammatory risk		Residual inflammatory risk	
	HR per SD (95% CI)	P-value	Adj. HR per SD (95% CI)	P-value	HR per SD (95% CI)	P-value	Adj. HR per SD (95% CI)	P-value
**FAI Score$_{CCTA}$ – Mean**	1.26 (1.23–1.29)	< .001	1.19 (1.16–1.23)	< .001	1.22 (1.20–1.25)	< .001	1.16 (1.14–1.19)	< .001
**FAI Score$_{CCTA}$ – LAD**	1.24 (1.21–1.27)	< .001	1.19 (1.16–1.23)	< .001	1.21 (1.18–1.23)	< .001	1.16 (1.14–1.19)	< .001
**FAI Score$_{CCTA}$ – LCX**	1.27 (1.22–1.31)	< .001	1.19 (1.14–1.23)	< .001	1.23 (1.20–1.26)	< .001	1.15 (1.12–1.18)	< .001
**FAI Score$_{CCTA}$ – RCA**	1.24 (1.21–1.28)	< .001	1.22 (1.18–1.26)	< .001	1.20 (1.17–1.22)	< .001	1.17 (1.15–1.20)	< .001
**FAI Score$_{NC}$- Mean**	1.28 (1.24–1.31)	< .001	1.20 (1.16–1.24)	< .001	1.24 (1.22–1.26)	< .001	1.17 (1.15–1.20)	< .001
**FAI Score$_{NC}$ – LAD**	1.26 (1.23–1.30)	< .001	1.19 (1.16–1.23)	< .001	1.23 (1.21–1.25)	< .001	1.17 (1.15–1.19)	< .001
**FAI Score$_{NC}$ – LCX**	1.29 (1.24–1.34)	< .001	1.19 (1.14–1.24)	< .001	1.24 (1.21–1.28)	< .001	1.15 (1.12–1.19)	< .001
**FAI Score$_{NC}$ – RCA**	1.31 (1.27–1.36)	< .001	1.25 (1.20–1.29)	< .001	1.26 (1.23–1.29)	< .001	1.19 (1.16–1.23)	< .001

HRs are expressed per 1 SD increment in the FAI Score as a continuous variable. Abbreviations: Adj. HR, hazard ratios further adjusted for coronary calcium score, and cardiovascular risk factors including age, sex, diabetes, hypertension, hyperlipidemia, smoking and CADRADS stenosis severity; CAD, Coronary artery disease; FAI ScoreCCTA, Fat attenuation index measured from coronary CT angiography; FAI ScoreNC, Fat attenuation index from non-contrast CT; HR, hazard ratios; LAD, left anterior descending coronary artery; LCX, left circumflex artery; MACE, major adverse cardiac events; RCA, right coronary artery.

© 2026 Chan K et al; ORFAN Consortium. *JAMA Cardiology*.

eTable 4. Predictive Value of FAI Score$_{CCTA}$ and FAI Score$_{NC}$ per Coronary Artery, for Myocardial Infarction, New Heart Failure

	Myocardial infarction				New-onset heart failure			
	**Total inflammatory risk HR (95% CI)**	**P-value**	**Residual inflammatory risk Adj. HR (95% CI)**	**P- value**	**Total inflammatory risk HR (95% CI)**	**P- value**	**Residual inflammatory risk Adj. HR (95% CI)**	**P- value**
**FAI Score$_{CCTA}$ – Mean**	1.16 (1.11–1.21)	< .001	1.06 (1.01–1.11)	0.019	1.25 (1.22–1.28)	< .001	1.16 (1.13–1.20)	< .001
**FAI Score$_{CCTA}$ – LAD**	1.14 (1.10–1.19)	< .001	1.07 (1.02–1.13)	0.003	1.23 (1.20–1.26)	< .001	1.17 (1.14–1.21)	< .001
**FAI Score$_{CCTA}$ – LCX**	1.13 (1.07–1.19)	< .001	1.00 (0.94–1.06)	0.99	1.26 (1.22–1.30)	< .001	1.15 (1.10–1.19)	< .001
**FAI Score$_{CCTA}$ – RCA**	1.15 (1.10–1.19)	< .001	1.07 (1.02–1.13)	0.011	1.21 (1.18–1.25)	< .001	1.16 (1.12–1.20)	< .001
**FAI Score$_{NC}$ –Mean**	1.15 (1.10–1.20)	< .001	1.03 (0.97–1.09)	0.351	1.27 (1.24–1.31)	< .001	1.19 (1.15–1.22)	< .001
**FAI Score$_{NC}$ –LAD**	1.14 (1.09–1.20)	< .001	1.06 (1.01–1.11)	0.029	1.26 (1.23–1.29)	< .001	1.19 (1.16–1.22)	< .001
**FAI Score$_{NC}$ –LCX**	1.14 (1.09–1.20)	< .001	0.98 (0.92–1.05)	0.552	1.29 (1.25–1.33)	< .001	1.16 (1.11–1.21)	< .001
**FAI Score$_{NC}$ –RCA**	1.15 (1.10–1.21)	< .001	1.02 (0.96–1.09)	0.445	1.30 (1.26–1.34)	< .001	1.20 (1.16–1.25)	< .001

HRs are expressed per 1 SD increment in the FAI Score as a continuous variable. Abbreviations: Adj. HR, hazard ratios further adjusted for coronary calcium score, and cardiovascular risk factors including age, sex, diabetes, hypertension, hyperlipidemia, smoking and CADRADS stenosis severity; FAI Score$_{CCTA}$, Fat attenuation index measured from coronary CT angiography; FAI Score$_{NC}$, Fat attenuation index from non-contrast CT; HR, hazard ratios; LAD, left anterior descending coronary artery; LCX, left circumflex artery; RCA, right coronary artery.

© 2026 Chan K et al; ORFAN Consortium. *JAMA Cardiology*.

eTable 5. Competing Risk Analysis for Cardiovascular Mortality

	Cause-specific Cox model		Fine–Gray competing risks model	
	**HR (95% CI)**	**P-value**	**SHR (95% CI)**	**P-value**
**FAI Score$_{CCTA}$ – Mean**	1.26 (1.23–1.29)	< .001	1.26 (1.19–1.34)	< .001
**FAI Score$_{CCTA}$ – LAD**	1.24 (1.21–1.27)	< .001	1.24 (1.18–1.30)	< .001
**FAI Score$_{CCTA}$ – LCX**	1.27 (1.22–1.31)	< .001	1.26 (1.21–1.31)	< .001
**FAI Score$_{CCTA}$ – RCA**	1.24 (1.21–1.28)	< .001	1.24 (1.19–1.29)	< .001
**FAI Score$_{NC}$ – Mean**	1.28 (1.24–1.31)	< .001	1.27 (1.20–1.36)	< .001
**FAI Score$_{NC}$ – LAD**	1.26 (1.23–1.30)	< .001	1.26 (1.18–1.34)	< .001
**FAI Score$_{NC}$ – LCX**	1.29 (1.24–1.34)	< .001	1.28 (1.22–1.34)	< .001
**FAI Score$_{NC}$ – RCA**	1.31 (1.27–1.36)	< .001	1.31 (1.24–1.38)	< .001

Cause-specific hazard ratios (HRs) were estimated using Cox proportional hazards regression. Subdistribution hazard ratios (SHRs) were estimated using Fine–Gray competing risks regression treating non-cardiovascular death as the competing event. Abbreviations: FAI Score$_{CCTA}$, Fat attenuation index measured from coronary CT angiography; FAI Score$_{NC}$, Fat attenuation index from non-contrast CT; LAD, left anterior descending coronary artery; LCX, left circumflex artery; RCA, right coronary artery.

© 2026 Chan K et al; ORFAN Consortium. *JAMA Cardiology*.

eTable 6. Cardiovascular Mortality Events per Risk Category

eTable 6. Cardiovascular mortality events per risk category

	Risk category based on CACS	Risk categories after addition of FAI Score$_{CCTA}$: <25th centile	25th–75th centile	75th–89th centile	≥90th centile	Total	Events correctly reclassified upward, No. (%)	Non-events correctly reclassified downward, No. (%)
	**No. of individuals experiencing an event /No. of all individuals**							
FAI Score$_{CCTA}$	CAC 0	11 /2866	37 /4332	15 /963	26 /490	89 /8651	41 (46.1)	7150 (83.5)
	CAC 1–99	21 /1271	54 /3112	23 /973	40 /607	138 /5963	63 (45.7)	4308 (74.0)
	CAC 100–299	9 /316	31 /1014	11 /422	32 /328	83 /2080	43 (51.8)	1290 (64.6)
	CAC ≥300	22 /328	66 /1103	55 /503	83 /482	226 /2416	138 (61.1)	1343 (61.3)
	Total	63 /4781	188 /9561	104 /2861	181 /1907	536/ 19110		
FAI Score$_{NC}$	CAC 0	17 /2677	32 /4420	21 /1054	19 /500	89 /8651	40 (44.9)	7048 (82.3)
	CAC 1–99	19 /1322	58 /3045	34 /966	27 /630	138 /5963	61 (44.2)	4290 (73.6)
	CAC 100–299	11 /389	33 /997	11 /370	28 /324	83 /2080	39 (47.0)	1342 (67.2)
	CAC ≥300	31 /390	80 /1093	50 /476	65 /457	226 /2416	115 (50.9)	1372 (62.6)
	Total	78 /4778	203 /9555	116 /2866	139 /1911	536 /19110		

© 2026 Chan K et al; ORFAN Consortium. *JAMA Cardiology*.

eReferences.

1. Chan K, Wahome E, Tsiachristas A, et al. Inflammatory risk and cardiovascular events in patients without obstructive coronary artery disease: the ORFAN multicentre, longitudinal cohort study. *Lancet*. Jun 15 2024;403(10444):2606-2618. doi:10.1016/s0140-6736(24)00596-8
2. Oikonomou EK, Antonopoulos AS, Schottlander D, et al. Standardized measurement of coronary inflammation using cardiovascular computed tomography: integration in clinical care as a prognostic medical device. *Cardiovasc Res*. Nov 22 2021;117(13):2677-2690. doi:10.1093/cvr/cvab286
3. Sobirov I, Xie C, Siddique M, et al. Structurally Different Neural Network Blocks for the Segmentation of Atrial and Aortic Perivascular Adipose Tissue in Multi-centre CT Angiography Scans. *Lect Notes Comput Sc*. 2026;15918:317-331. doi:10.1007/978-3-031-98694-9_23

© 2026 Chan K et al; ORFAN Consortium. *JAMA Cardiology*.
