# Supplementary material for: Assessment of Coronary Inflammatory Risk From Noncontrast Cardiac Computed Tomography
Source: JAMA Cardiol. 2026 Nov 11:e265006. Online ahead of print. doi: 10.1001/jamacardio.2026.5006 (PMC13756199; doi:10.1001/jamacardio.2026.5006)
Supplement: Supplementary file 2 [file jamacardiol-e265006-s002.pdf]

Supplemental Online Content: Nonauthor Collaborators
*First name, last name, and suffix (if applicable) are required and will appear in PubMed.

***Group Name(s): ORFAN Consortium**

***First Name and Middle Initial(s)**	***Last Name**	***Suffix (eg, Jr, III)**	Academic Degrees	Institution	Location (city, state/province, country)	Role or Contribution, eg, chair, principal investigator	Group (if more than 1 Group listed in the byline) and/or Subgroup (eg, Steering Committee)
Sheena	Thomas			ORFAN core laboratory, Acute Multidisciplinary Imaging and Interventional Centre, Division of Cardiovascular Medicine, Radcliffe Department of Medicine, University of Oxford	Oxford, UK		
Jon	Denton			ORFAN core laboratory, Acute Multidisciplinary Imaging and Interventional Centre, Division of Cardiovascular Medicine, Radcliffe Department of Medicine, University of Oxford	Oxford, UK		
Robyn	Farrall			ORFAN core laboratory, Acute Multidisciplinary Imaging and Interventional Centre, Division of Cardiovascular Medicine, Radcliffe Department of Medicine, University of Oxford	Oxford, UK		
Leon	Dong			ORFAN core laboratory, Acute Multidisciplinary Imaging and Interventional Centre, Division of Cardiovascular Medicine, Radcliffe Department of Medicine, University of Oxford	Oxford, UK		

*First name, last name, and suffix (if applicable) are required and will appear in PubMed.

***First Name and Middle Initial(s)**	***Last Name**	***Suffix (eg, Jr, III)**	Academic Degrees	Institution	Location (city, state/province, country)	Role or Contribution, eg, chair, principal investigator	Group (if more than 1 Group listed in the byline) and/or Subgroup (eg, Steering Committee)
Athanasia	Panopoulou			ORFAN core laboratory, Acute Multidisciplinary Imaging and Interventional Centre, Division of Cardiovascular Medicine, Radcliffe Department of Medicine, University of Oxford	Oxford, UK		
Karolina	Rehakova			ORFAN core laboratory, Acute Multidisciplinary Imaging and Interventional Centre, Division of Cardiovascular Medicine, Radcliffe Department of Medicine, University of Oxford	Oxford, UK		
Mary	Kasongo			ORFAN core laboratory, Acute Multidisciplinary Imaging and Interventional Centre, Division of Cardiovascular Medicine, Radcliffe Department of Medicine, University of Oxford	Oxford, UK		
Victoria	Pettitt			ORFAN core laboratory, Acute Multidisciplinary Imaging and Interventional Centre, Division of Cardiovascular Medicine, Radcliffe Department of Medicine, University of Oxford	Oxford, UK		

Supplemental Online Content: Nonauthor Collaborators
*First name, last name, and suffix (if applicable) are required and will appear in PubMed.

***First Name and Middle Initial(s)**	***Last Name**	***Suffix (eg, Jr, III)**	Academic Degrees	Institution	Location (city, state/province, country)	Role or Contribution, eg, chair, principal investigator	Group (if more than 1 Group listed in the byline) and/or Subgroup (eg, Steering Committee)
Andy	Marfleet			ORFAN core laboratory, Acute Multidisciplinary Imaging and Interventional Centre, Division of Cardiovascular Medicine, Radcliffe Department of Medicine, University of Oxford	Oxford, UK		
Chrisha	Ledesma			ORFAN core laboratory, Acute Multidisciplinary Imaging and Interventional Centre, Division of Cardiovascular Medicine, Radcliffe Department of Medicine, University of Oxford	Oxford, UK		
Cheng	Xie			ORFAN core laboratory, Acute Multidisciplinary Imaging and Interventional Centre, Division of Cardiovascular Medicine, Radcliffe Department of Medicine, University of Oxford	Oxford, UK		
Bruno	Silva Santos			ORFAN core laboratory, Acute Multidisciplinary Imaging and Interventional Centre, Division of Cardiovascular Medicine, Radcliffe Department of Medicine, University of Oxford	Oxford, UK		

Supplemental Online Content: Nonauthor Collaborators
*First name, last name, and suffix (if applicable) are required and will appear in PubMed.

*First Name and Middle Initial(s)	*Last Name	*Suffix (eg, Jr, III)	Academic Degrees	Institution	Location (city, state/province, country)	Role or Contribution, eg, chair, principal investigator	Group (if more than 1 Group listed in the byline) and/or Subgroup (eg, Steering Committee)
Michail C	Mavrogiannis			ORFAN core laboratory, Acute Multidisciplinary Imaging and Interventional Centre, Division of Cardiovascular Medicine, Radcliffe Department of Medicine, University of Oxford	Oxford, UK		
Susan	Anthony			Oxford University Hospitals NHS Foundation Trust	Oxford, UK		
Adrian	Banning			Oxford University Hospitals NHS Foundation Trust	Oxford, UK		
Rafail A	Kotronias			ORFAN core laboratory, Acute Multidisciplinary Imaging and Interventional Centre, Division of Cardiovascular Medicine, Radcliffe Department of Medicine, University of Oxford	Oxford, UK		
Tarun K	Mittal			Royal Brompton and Harefield Hospitals	London, UK		
Anne	Rose			Milton Keynes University Hospital NHS Foundation Trust	Milton Keynes, UK		
George	Hudson			Department of Cardiovascular Sciences and National Institute of Health Research Leicester Biomedical Research Centre, University of Leicester	Leicester, UK		
Amrita	Bajaj			Department of Cardiovascular Sciences and National Institute of Health Research Leicester Biomedical Research Centre, University of Leicester	Leicester, UK		

Supplemental Online Content: Nonauthor Collaborators
*First name, last name, and suffix (if applicable) are required and will appear in PubMed.

***First Name and Middle Initial(s)**	***Last Name**	***Suffix (eg, Jr, III)**	Academic Degrees	Institution	Location (city, state/province, country)	Role or Contribution, eg, chair, principal investigator	Group (if more than 1 Group listed in the byline) and/or Subgroup (eg, Steering Committee)
Intrajeet	Das			Department of Cardiovascular Sciences and National Institute of Health Research Leicester Biomedical Research Centre, University of Leicester	Leicester, UK		
Aparna	Deshpande			Department of Cardiovascular Sciences and National Institute of Health Research Leicester Biomedical Research Centre, University of Leicester	Leicester, UK		
Praveen	Rao			Department of Cardiovascular Sciences and National Institute of Health Research Leicester Biomedical Research Centre, University of Leicester	Leicester, UK		
Dan	Lawday			Department of Cardiovascular Sciences and National Institute of Health Research Leicester Biomedical Research Centre, University of Leicester	Leicester, UK		
Saeed	Mirsadraee			Royal Brompton and Harefield NHS Foundation Trust	London, UK		
Benjamin	Hudson			Royal United Hospitals Bath NHS Foundation Trust and Department of Health, University of Bath,	Bath, UK		
John	Graby			Royal United Hospitals Bath NHS Foundation Trust and Department of Health, University of Bath,	Bath, UK		
David	Murphy			Royal United Hospitals Bath NHS Foundation Trust and Department of Health, University of Bath,	Bath, UK		
Colin	Berry			NHS Greater Glasgow and Clyde	Glasgow, UK		

Supplemental Online Content: Nonauthor Collaborators
*First name, last name, and suffix (if applicable) are required and will appear in PubMed.

***First Name and Middle Initial(s)**	***Last Name**	***Suffix (eg, Jr, III)**	Academic Degrees	Institution	Location (city, state/province, country)	Role or Contribution, eg, chair, principal investigator	Group (if more than 1 Group listed in the byline) and/or Subgroup (eg, Steering Committee)
Mohamed	Marwan			Department of Cardiology, Friedrich-Alexander-Universität Erlangen-Nürnberg	Erlangen, Germany		
Pál	Maurovich-Horvat			Department of Radiology, MTA-SE Cardiovascular Imaging Research Group	Budapest, Hungary		
Guo-Wei	He			TEDA International Cardiovascular Hospital	Tianjin, China		
Wen-Hua	Lin			TEDA International Cardiovascular Hospital	Tianjin, China		
Li-Juan	Fan			TEDA International Cardiovascular Hospital	Tianjin, China		
Naohiko	Takahashi			Oita University	Japan		
Hidekazu	Kondo			Oita University	Japan		
Neng	Dai			Fudan University	Shanghai, China		
Junbo	Ge			Fudan University	Shanghai, China		
Bon-Kwon	Koo			Seoul National University	Seoul, South Korea		
Marco	Guglielmo			Centro Cardiologico Monzino IRCCS, University of Milan	Milan, Italy		
Gianluca	Pontone			Centro Cardiologico Monzino IRCCS, University of Milan	Milan, Italy		
Theodora	Benedek			University of Medicine and Pharmacy of Tirgu Mures	Romania		
Dijana	Vilic			Guy's and St Thomas' NHS Foundation Trust	London, UK		
Haleema	Aljazzaf			Guy's and St Thomas' NHS Foundation Trust	London, UK		
Mak S	Mun			Guy's and St Thomas' NHS Foundation Trust	London, UK		

Supplemental Online Content: Nonauthor Collaborators
*First name, last name, and suffix (if applicable) are required and will appear in PubMed.

***First Name and Middle Initial(s)**	***Last Name**	***Suffix (eg, Jr, III)**	Academic Degrees	Institution	Location (city, state/province, country)	Role or Contribution, eg, chair, principal investigator	Group (if more than 1 Group listed in the byline) and/or Subgroup (eg, Steering Committee)
Giulia	Benedetti			Guy's and St Thomas' NHS Foundation Trust	London, UK		
Rebecca L	Preston			Guy's and St Thomas' NHS Foundation Trust	London, UK		
Zahra	Raisi-Estabragh			Queen Mary University of London	London, UK		
Derek L	Connolly			Sandwell and West Birmingham Hospitals NHS Trust	Birmingham, UK		
Rebecca	Grenfell			Sandwell and West Birmingham Hospitals NHS Trust	Birmingham, UK		
William	Bradlow			University Hospitals Birmingham (UHB) NHS Trust	Birmingham, UK		
Matthias	Schmitt			University Hospital of Manchester Foundation Trust	Manchester, UK		
Fabiano	Serfaty			Serfaty Clinicas, Rio de Janeiro	Brazil		
Ilan	Gottlieb			Casa de Saúde São José, Rio de Janeiro	Brazil		
Mario FT	Neves			Universidade do Estado do Rio de Janeiro	Rio de Janeiro, Brazil		
David E	Newby			University of Edinburgh	Edinburgh, UK		
Tomasz	Guzik			University of Edinburgh	Edinburgh, UK		
Marc R	Dweck			University of Edinburgh	Edinburgh, UK		
Guido	Claessen			KU Leuven	Lueven, Belgium		
Stéphane	Hatem			Foundation for Innovation in Cardiometabolism and Nutrition (ICAN)	Paris, France		
Louise	Meyfroit			Foundation for Innovation in Cardiometabolism and Nutrition (ICAN)	Paris, France		
Alban	Redheuil			Sorbonne Université, Faculté de Médecine Pierre et Marie Curie	Paris, France		
Georgios	Benetos			Lefkos Stavros Clinic Athens	Greece		
Meinrad	Beer			Universitätsklinikum Ulm	Germany		

Supplemental Online Content: Nonauthor Collaborators
*First name, last name, and suffix (if applicable) are required and will appear in PubMed.

***First Name and Middle Initial(s)**	***Last Name**	***Suffix (eg, Jr, III)**	Academic Degrees	Institution	Location (city, state/province, country)	Role or Contribution, eg, chair, principal investigator	Group (if more than 1 Group listed in the byline) and/or Subgroup (eg, Steering Committee)
Gastón A	Rodriguez-Granillo			ENERI Medical Institute	Argentina		
Joseph	Selvanayagam			South Australia Health and Medical Research Institute	Adelaide, Australia		
Francisco	Lopez-Jimenez			Mayo Clinic	Rochester, USA		
Ruben	De Bosscher			University Hospitals Leuven	Belgium		
Alain	Tavildari			Cardiovista	France		
Gemma	Figtree			University of Sydney	Australia		
Stuart Michael	Grieve			University of Sydney	Sydney, Australia		
Ibrahim	Danad			Amsterdam University Medical Centers	Netherlands		
Ronney	Shantouf			Cleveland Clinic Abu Dhabi	UAE		
Bas	Kietselaer			Zuyderland Medical Centre	Heerlen, Netherlands		
Dimitris	Tousoulis			University of Athens	Greece		
George	Dangas			Mount Sinai School of Medicine	New York, USA		
Leslee	Shaw			Mount Sinai Hospital	New York, USA		
Shahzad	Munir			Heart and Lung Centre, New Cross Hospital, Wolverhampton	Wolverhampton, UK		
Elisa	McAlindon			Heart and Lung Centre, New Cross Hospital, Wolverhampton	Wolverhampton, UK		
Marly	Van Assen			University of Emory	Atlanta, USA		
Filippo	Crea			Isola Tiberina-Gemelli Isola Hospital	Rome, Italy		
Giulia	La Vecchia			Isola Tiberina-Gemelli Isola Hospital	Rome, Italy		
Stephen A.	Bloom			Midwest Heart & Vascular Specialists LLC	Kansas city, USA		
Vasvi	Singh			Midwest Heart & Vascular Specialists LLC	Kansas city, USA		
Nick	Ho			Bridging Medical Silos	Tampa, USA		
Wang	Dee Dee			NCH-Rooney Heart Institute	Naples, USA		
Carl E.	Orringer			NCH-Rooney Heart Institute	Naples, USA		
Todd	Villines			University of Virginia	Charlottesville, USA		

Supplemental Online Content: Nonauthor Collaborators
*First name, last name, and suffix (if applicable) are required and will appear in PubMed.

***First Name and Middle Initial(s)**	***Last Name**	***Suffix (eg, Jr, III)**	Academic Degrees	Institution	Location (city, state/province, country)	Role or Contribution, eg, chair, principal investigator	Group (if more than 1 Group listed in the byline) and/or Subgroup (eg, Steering Committee)
Jessica	Lee			University of Virginia	Charlottesville, USA		
Lora	Nordstrom			Valleywise Health Medical Center	Phoenix, USA		
Claudia	Islas			Valleywise Health Medical Center	Phoenix, USA		
Erika	Hutt			St Lukes Hospital	Kansas city, USA		
Hsiang-Chun	Lee			Taiwan Kaohsiung Medical University	Kaohsiung, Taiwan		
Simon	Winther			Department of Cardiology, Gødstrup Hospital, Department of Clinical Medicine, Aarhus University	Aarhus, Danmark		
Nikolaos	Kadoglou			Nicosia Polyclinic	Nicosia, Cyprus		
Thijs	Eijsvogels			Radbound University Medical Center	Nijmegen, the Netherlands		
Thijs	Schrama			Radbound University Medical Center	Nijmegen, the Netherlands		
Vincent	Aengevaeren			Radbound University Medical Center	Nijmegen, the Netherlands		
