# Supplementary material for: Assessment of Coronary Inflammatory Risk From Noncontrast Cardiac Computed Tomography
Source: JAMA Cardiol. 2026 Nov 11:e265006. Online ahead of print. doi: 10.1001/jamacardio.2026.5006 (PMC13756199; doi:10.1001/jamacardio.2026.5006)
Supplement: Supplementary file 3 [file jamacardiol-e265006-s003.pdf]

Data Sharing Statement

Chan. Assessment of Coronary Inflammatory Risk From Noncontrast Cardiac Computed Tomography. *JAMA Cardiol*. Published November 11, 2026.
doi:10.1001/jamacardio.2026.5006

Data
Data available: No
